# A Systematic Literature Review of Health Utility Values in Breast Cancer

**DOI:** 10.1177/0272989X211065471

**Published:** 2022-01-18

**Authors:** Manraj N. Kaur, Jiajun Yan, Anne F. Klassen, Justin P. David, Dilshan Pieris, Manraj Sharma, Louise Bordeleau, Feng Xie

**Affiliations:** School of Rehabilitation Sciences, Faculty of Health Sciences, McMaster University, Hamilton, ON, Canada; Department of Health Research Methods, Evidence and Impact, Faculty of Health Sciences, McMaster University, Hamilton, ON, Canada; Department of Pediatrics, Faculty of Health Sciences, McMaster University, Hamilton, ON, Canada; Faculty of Medicine, University of Toronto, Toronto, ON, Canada; Faculty of Medicine, University of Toronto, Toronto, ON, Canada; Faculty of Medicine, University of Toronto, Toronto, ON, Canada; Department of Oncology, Division of Medical Oncology, Faculty of Health Sciences, McMaster University, Hamilton, ON, Canada; Department of Health Research Methods, Evidence and Impact, Faculty of Health Sciences, McMaster University, Hamilton, ON, Canada

**Keywords:** breast cancer, economic evaluation, meta-regression, health status, health states, health-related quality of life, health state utility values, health utilities, PRISMA, review, utility score

## Abstract

**Background:**

Health utility values (HUVs) are important inputs to the cost-utility analysis of breast cancer interventions.

**Purpose:**

Provide a catalog of breast cancer–related published HUVs across different stages of breast cancer and treatment interventions.

**Data Sources:**

Systematic searches of MEDLINE, MEDLINE In-Process, EMBASE, Web of Science, CINAHL, PsycINFO, EconLit, and Cochrane databases (2005–2017).

**Study Selection:**

Studies published in English that reported mean or median HUVs using direct or indirect methods of utility elicitation for breast cancer.

**Data Extraction:**

Independent reviewers extracted data on a preestablished and piloted form; disagreements were resolved through discussion.

**Data Analysis:**

Mixed-effects meta-regression using restricted maximum likelihood modeling was conducted for intervention type, stage of breast cancer, and typical clinical and treatment trajectory of breast cancer patients to assess the effect of study characteristics (i.e., sample size, utility elicitation method, and respondent type) on HUVs.

**Data Synthesis:**

Seventy-nine studies were included in the review. Most articles (*n* = 52, 66%) derived HUVs using the EQ-5D. Patients with advanced-stage breast cancer (range, 0.08 to 0.82) reported lower HUVs as compared with patients with early-stage breast cancer (range, 0.58 to 0.99). The meta-regression analysis found that undergoing chemotherapy and surgery and radiation, being diagnosed with an advanced stage of breast cancer, and recurrent cancer were associated with lower HUVs. The members of the general public reported lower HUVs as compared with patients.

**Limitations:**

There was considerable heterogeneity in the study population, health states assessed, and utility elicitation methods.

**Conclusion:**

This review provides a catalog of published HUVs related to breast cancer. The substantial heterogeneity in the health utility studies makes it challenging for researchers to choose which HUVs to use in cost-utility analyses for breast cancer interventions.

## Introduction

Patient-centered decisions about breast cancer treatments often involve tradeoffs between the possible benefits and harms. Such tradeoffs are personal judgments that may differ among individuals; some women may judge that the survival benefits of cancer treatment outweigh the potential toxicity, while others may place greater value on health-related quality of life (HRQOL) over survival. The measure of quality-adjusted life-years (QALYs) combines both survival and impact on HRQOL. The HRQOL impact (the “Q”) in a QALY is measured by health utilities.^1^ Health utilities are cardinal values that represent the strength of an individual’s preferences for the health outcome or health state under consideration.^2,3^ Hence, a more desirable health outcome will have higher health utility value and vice versa. Health utilities are anchored at 0 for death and 1 for full health or the best possible outcome. Health states that are considered worse than death are indicated by negative values.^3^ In breast cancer, health utilities have been measured using direct utility elicitation methods such as standard gamble (SG), time tradeoff (TTO), or rating scales or using indirect methods with self-reported, generic preference-based instruments such as the EQ-5D,^4^ the Short Form–6D (SF-6D),^5,6^ and the Health Utilities Index Mark 3 (HUI3).^7^

The health utility values for the same health outcome or health state can vary substantially depending on the method of health utility estimation, the population used to derive utility scores (patients, caregivers, health professionals, or the general public), and the context (setting, method or mode of administration, or description of health state). This heterogeneity in the health utility values makes it challenging for researchers to choose which values to use for the calculation of QALY in cost-utility analyses. A previous systematic review by Peasgood et al.^8^ summarized published utilities in breast cancer and pooled utilities for some breast cancer–related health states for peer-reviewed studies published up to 2007. Peasgood et al.^8^ concluded that because of substantial variation in the method of utility estimation and the source of utility values in breast cancer, the pooling of most utility values was problematic. To the best of our knowledge, no comprehensive systematic review of the breast cancer literature has been conducted since then. Hence, the objective of this systematic review of the literature was to identify and descriptively summarize the published health utility values related to breast cancer. The scope of this review covers the full spectrum of cancer care, ranging from screening to palliative care, whenever reported.

## Methods

### Search Strategy

A review of the literature published between January 1, 2005 and August 2017 was conducted. This timeline was chosen to ensure that the included health utilities are relevant to the current diagnostic and treatment guidelines. The electronic databases of MEDLINE, MEDLINE In-Process, EMBASE, Web of Science, CINAHL, PsycINFO, EconLit, and Cochrane databases (Cochrane Database of Systematic Reviews, Database of Abstracts of Reviews of Effectiveness [DARE], Cochrane Central Register of Clinical Trials, Health Technology Assessment [HTA], and NHS Economic Evaluation Database [NHS EED]) were searched. The electronic search strategy, designed with the help of a medical librarian, used health utility and utility elicitation method-specific terms, combined with breast cancer. The database search was complemented with a bibliographic hand search of citations included in the articles that met the study inclusion criteria. The search strategy is provided in the supplementary material.

### Study Eligibility

The studies were screened in 2 phases. In phase 1, the titles and abstracts retrieved from the electronic databases search were reviewed by 1 author (M.N.K.). Studies in which the title or the abstract clearly indicated that the health utility values were elicited for adult patients with breast cancer were included for the phase 2 screening. We excluded literature reviews, meta-analyses, psychometric evaluations, editorials, comment letters, animal studies, conference abstracts, studies published in languages other than English, and studies in which health utility values were obtained from the literature.

In the phase 2 screening, full texts of the studies that met the inclusion criteria in phase 1 were reviewed by 2 independent reviewers (M.N.K. and P.D., J.P.D., or M.S.) using a predetermined screening form that was piloted using 5 studies. Studies were included if they 1) reported health utility values for adult breast cancer patients, including treatment-related and adverse events, and 2) described methods of utility assessment. Interreviewer disagreements were resolved through discussion, and a senior author (F.X.) was consulted if the disagreement persisted.

### Data Extraction and Management

The data from the included studies were extracted onto a predesigned data extraction form, which was piloted with 5 articles. The data extraction was completed by 2 reviewers independently (M.K. and J.P.D., D.P., or M.S.). The following variables were recorded: 1) first listed author, publication year, country, journal, and funding source; 2) study design; 3) number and type of respondents from whom utilities were elicited (i.e., patients or nonpatients); 4) method of utility elicitation (direct or indirect); for direct studies, data on whether pilot testing was completed, whether interviewer was trained, and whether inconsistencies were assessed and recorded; for indirect studies, data on the country of scoring algorithm (where provided) were recorded; 5) administration method; and 6) reported mean or median utility, with variance (where provided). Disutilities were converted to utilities for the purposes of consistency in reporting and analyses.

A thematic approach was adopted for data management whereby the health utilities extracted from articles were classified into 2 main categories: 1) intervention-specific utilities and 2) breast cancer stage–specific utilities. Intervention-specific health utility values were further organized into 1) screening, 2) noninvasive and invasive diagnostic procedures, 3) local therapy (i.e., radiation or/and surgery), 4) systemic therapy (i.e., chemotherapy, endocrine therapy, targeted therapy), 5) allied health and complementary medicine, and 6) adverse events and their treatments. Breast cancer stage–specific health utility values were organized into 1) early breast cancer, 2) advanced or metastatic breast cancer, and 3) nonspecific breast cancer for when the stage of breast cancer was not specified or could not be ascertained from the article.

Descriptive analyses were completed to summarize the results. To assess the effect of study characteristics (i.e., sample size, valuation method, and type of respondent) on utility values, a meta-regression analysis was performed with utility value as the dependent variable and the study characteristics as independent variables. The heterogeneity of variance was estimated by fitting 2 separate mixed-effects models for type of intervention and stage of breast cancer using restricted maximum likelihood. The 2 models were in alignment with the thematic approach of the paper (i.e., describing health utilities by intervention and stage of breast cancer). Combining these 2 models into 1 model was deemed inappropriate, as it would have resulted in substantial heterogeneity, potentially rendering the model estimates unusable. For the purposes of meta-regression analysis, the type of intervention was categorized into screening, noninvasive diagnostic procedure, invasive diagnostic procedure, surgery, radiation, surgery and radiation, chemotherapy, and endocrine therapy. The stage of breast cancer was categorized into early, advanced or metastatic, and nonspecific breast cancer. The valuation methods were organized into EQ-5D, SG, TTO, visual analog scale (VAS), and others. Respondents were organized into patients and public. For studies that reported multiple utility values for the same defined health states by intervention or stage, the utility values were averaged and treated as one record for the analysis. The meta-regression analysis was completed using R version 4.0.3 and the lme4 package.

## Results

### Review Process

**Figure 1:**
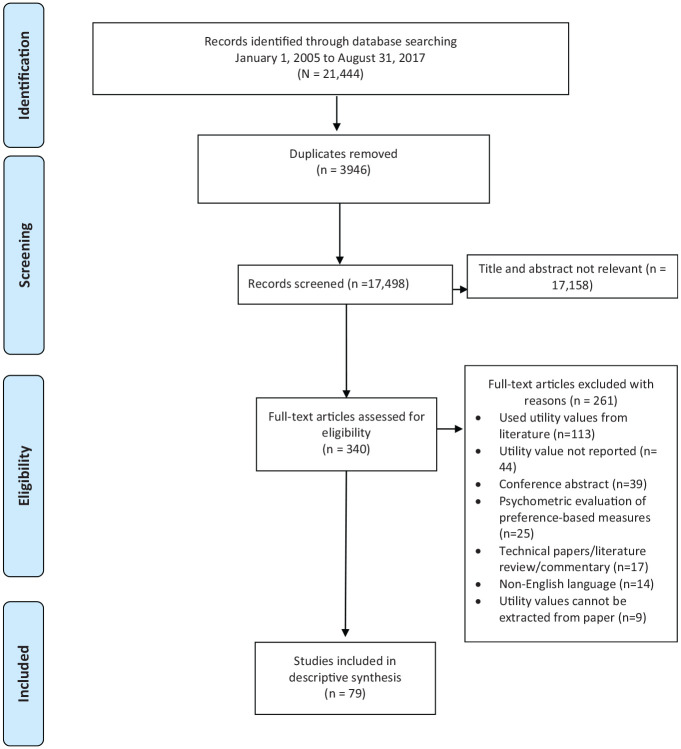
PRISMA flow diagram for the systematic literature review of published health utility values in breast cancer.

As shown in the PRISMA diagram (Figure 1), the electronic literature search yielded 21,444 records. Of these, 3,946 records were published in duplicate and were removed. After phase 1 screening, 17,158 records were excluded based on title and abstract screening. The remaining 340 full-text articles were retrieved and reviewed for inclusion. Of these, 79 were included in the review.

### Study Characteristics

A summary of the study characteristics are shown in Table 1 (for a detailed description of study characteristics, please see the supplementary material). Thirty-nine studies (49.4%) were published in oncology journals, followed by health economics and outcomes research journals (*n* = 22, 27.8%), and the remaining articles were published in other medical or public health journals (*n* = 18, 22.8%). A total of 37 articles (46.8%) received funding from not-for-profit or academic sources, 18 from for-profit sources (22.8%), and 3 from a combination of the two (3.8%). The remaining 21 articles (26.6%) did not receive funding or disclose a funding source. The number of articles published per year gradually increased since 2005, with the highest number of articles (*n* = 12, 15.2%) published in 2017. The corresponding author(s) for most of the publications were based in the United States (*n* = 23, 29.1%), the Netherlands (*n* = 8, 10.1%), Australia (*n* = 6, 7.6%), and Canada (*n* = 6, 7.6%).

**Table 1:** Summary of Study Characteristics

Study Characteristic	*N*
Country of corresponding author	
United States	23
The Netherlands	9
United Kingdom	7
Canada	6
Australia	6
Japan	4
Sweden	4
Germany, Greece, Iran, Korea, Singapore, Spain	2 each
China, Finland, Lebanon, Malaysia, South Korea, Switzerland, Taiwan, Thailand	1 each
Study design for preference elicitation study	
Cross-sectional	46
Randomized controlled trial	16
Cohort, prospective	14
Cohort, retrospective	3
Respondents	
Patients	56
Public, women	11
Public	5
Public, women and patients	3
Public and patients	2
Health care professionals	2
Health utility elicitation method	
EQ-5D	51
SG	13
TTO	12
VAS	10
SF-6D	4
SHE	2
TMI	2
AQOL-4D, 15D, QOL VAS, VR-6D, HALex	1 each

AQOL, Australian Quality of Life; SF-6D, HALex, Health and Activities Limitation Index, Short Form-6D; SG, standard gamble; SHE, Subjective Health Estimation; TMI, Testing Morbidities Index; TTO, time tradeoff; QOL, quality of life; VAS, visual analog scale; VR-6D, Veterans RAND-6D.

Indirect methods using multiattribute utility instruments were more common than direct methods of utility estimation. Direct methods of utility estimation were used in 18 studies (22.8%), where SG was the most common approach, followed by TTO and VAS. Indirect methods were used in 55 studies (69.6%) and a combination of direct and indirect methods in 6 studies (7.6%). Of 18 articles reporting on direct studies, 7 (38.9%) studies piloted the methods prior to administration, 9 (50%) reported on using trained interviewers, and 7 (38.9%) assessed inconsistencies in responses and adjusted their analyses accordingly. The health states to be assessed were identified and defined using literature review (*n* = 10, 55.6%), consultation with health care professionals experienced in treating women with breast cancer (*n* = 8, 44.4%), interviews with women diagnosed with breast cancer (*n* = 4, 22.2%), published guidelines or medical labeling information (*n* = 3, 16.7%), epidemiological data (*n* = 1, 5.6%), a previously developed questionnaire (*n* = 1, 5.6%), and breast cancer web forums (*n* = 1, 5.6%). Six (33.3%) studies did not specify how health states were developed.

Of 55 studies that used the indirect methods, the EQ-5D-3L was the most common preference-based measure (*n* = 48, 87.3%), followed by the SF-6D (*n* = 4, 7.3%). The remaining studies used the Finnish 15D,^9^ HUI3,^10^ or Assessment of Quality of Life 4–dimension (AQOL-4D).^11^ Five studies mapped the data from EORTC-QLQ-C 30 to the EQ-5D-3L,^12–16^ and 1 study mapped the SF-12 to VR-6D^17^ using published algorithms. Three studies used the 5L version of the EQ-5D.^18–20^ Four studies (5.1%) compared direct and indirect methods,^21–24^ 6 studies (7.6%) compared 1 or more types of direct utility estimation methods,^20,21,25–28^ and 3 studies (3.8%) compared 1 or more types of indirect utility elicitation methods.^9,13,29^ Three studies (5.4%) compared country-specific algorithms.^19,21,30^

In terms of the respondents who completed the utility estimation exercise, most studies used women diagnosed with breast cancer (*n* = 57, 72.2%), followed by members of the general public (*n* = 14, 17.7%), a combination of the general public and women diagnosed with breast cancer (*n* = 6, 7.6%), and health care professionals (*n* = 2, 2.5%). The full list of breast cancer–relevant health states and the utilities are provided in the supplementary material.

### Intervention-Specific Health Utility Values

#### Screening or diagnostic interventions

**Figure 2:**
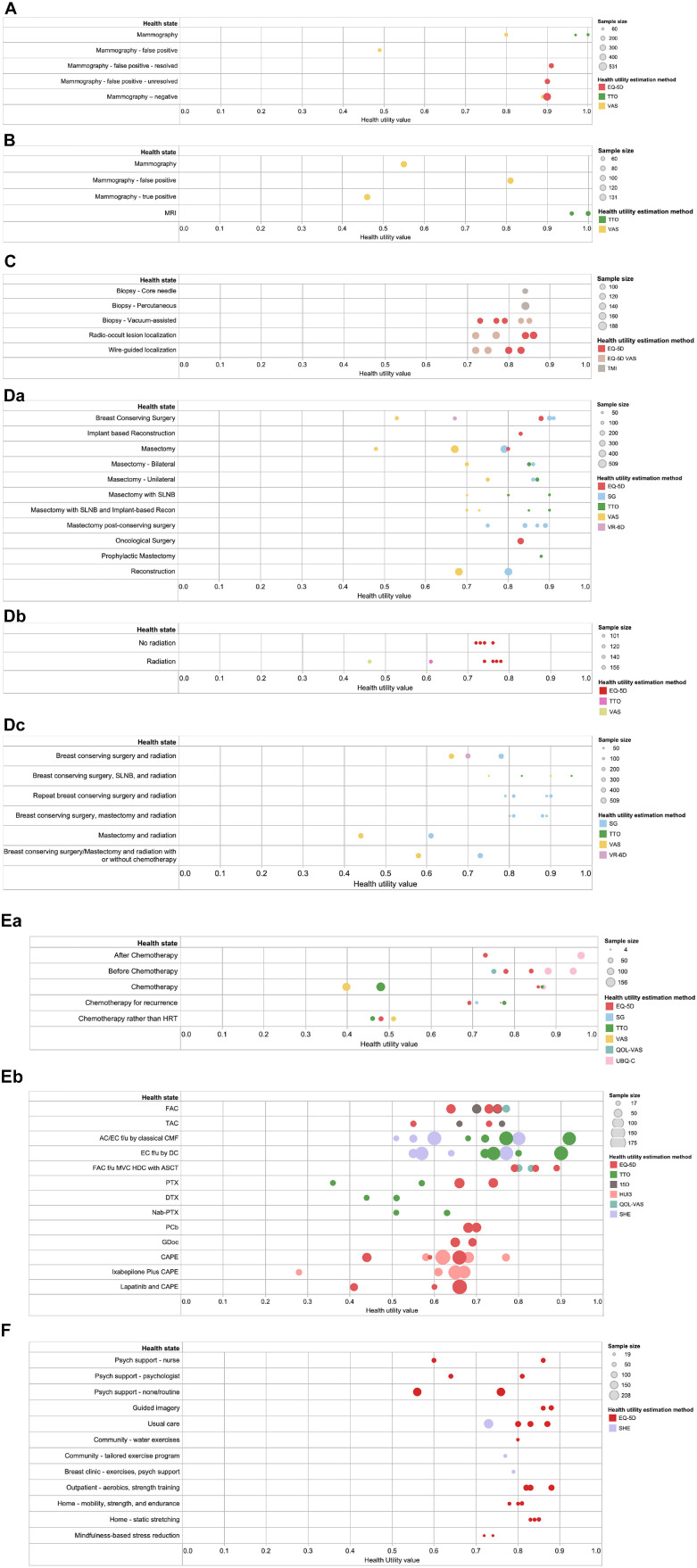
Health utility values in breast cancer, by treatment intervention. (a) Screening interventions. (b) Noninvasive diagnostic interventions. (c) Invasive diagnostic interventions. (d) Local interventions: a, surgery; b, radiation; c, radiation and surgery. (e) Systematic interventions: a, chemotherapy, drug not specified; b, chemotherapy, drugs specified. (f) Allied health or complementary medicine interventions.

Eight studies^55,62,64,78,79,83,84,89^ measured utility values for health states related to breast cancer screening or diagnostic interventions and are shown in Figure 2a-c. The mean values ranged from 0.46 to 1.00. Three mammography-related health states of false-positive results on screening mammography and receiving diagnostic mammography and a true-positive result on diagnostic mammography had lower mean utilities (range, 0.46 to 0.55) compared with the other health states (range, 0.72 to 1.00). Most of the health states were measured using either the EQ-5D or VAS, and others used the Testing Morbidities Index and TTO. The values obtained from the VAS were placed on the lower end of the 0–1 scale, the EQ-5D values were in the middle, and the TTO values fell on the higher end.

#### Local therapy

Thirteen studies^17,20,26,27,46,55,64,73,77,81,82,87,97^ used direct (SG, TTO, VAS) and indirect (EQ-5D, VR-6D) methods to obtain health utilities for breast cancer surgery (Figure 2b). Breast cancer surgery–related mean utilities were found to have large variation; utilities for breast-conserving surgery (range, 0.53 to 0.91) and mastectomy (range, 0.48 to 0.87) were found to be lower compared with utilities for mastectomy followed by breast-conserving surgery (range, 0.75 to 0.89) and bilateral mastectomy (range, 0.70 to 0.86). Breast reconstruction–related utilities were found to be between 0.68 and 0.90. The mean utilities derived from VAS tended to be lower (range, 0.48 to 0.80) than those from other methods, which clustered between 0.67 and 0.91.

Four studies^48,52,55,87^ measured utilities for radiation as compared with no radiation. The sample size of the studies was similar, and the median utilities for no radiation were slightly higher as compared with radiation. The utilities derived using the VAS were found to be on the lower end, TTO fell in the middle, and the EQ-5D values were on the higher end.

Five studies^17,20,46,63,97^ measured utilities related to breast cancer surgery and radiation combined. The values for mastectomy and radiation were lowest (range, 0.44 to 0.61), whereas the utilities for breast-conserving surgery (with or without mastectomy) and radiation or repeat breast-conserving surgery and radiation fell between 0.66 and 0.95. The utilities derived from VAS were at the lower end (range, 0.44 to 0.90) as compared with SG, TTO, and VR-6D. Most of the utilities in this category were derived from SG (range, 0.61 to 0.90).

#### Systemic therapy

The utilities associated with chemotherapy were categorized into when no drug was specified (*n* = 9)^21,23,24,48,51,55,80,87,90^ or drugs were specified (*n* = 11)^10,13,23,54,56,58-60,70,85,92^. When no drug was specified, the utilities for chemotherapy health states (for primary and recurrence) were found to be lower and had much larger variability (range, 0.40 to 0.92) as compared with those before and after chemotherapy (range, 0.73 to 0.84). The utilities derived from the VAS were on the lower end. When the drug or drug combination for chemotherapy-related utilities was specified, substantial variation in the values was observed (Figure 2c). Utilities ranged from 0.28 to 0.92 and were measured using primarily self-reported instruments, the EQ-5D, Finnish 15D, HUI3, QOL-VAS, and Subjective Health Estimation.

Ten studies^21,24,48,51,55,57,64,71,87,94^ measured endocrine therapy–related utilities. The utilities for nonspecific hormone replacement therapy ranged from 0.52 to 0.93, whereas for tamoxifen, the utilities were on the higher end and ranged from 0.75 to 0.95. One study with a sample size of 152 patients^71^ assessed the utility for goserelin therapy using SG and reported a mean utility value of 0.81. None of the identified studies reported utilities for targeted therapies for breast cancer.

#### Allied health and complementary medicine

Ten studies^14,45,61,65,67,74,86,95,96,98^ assessed utilities associated with allied health and complementary medicine–related health states (Figure 2d). Most of the studies used the EQ-5D, and the mean utility values ranged from 0.56 to 0.88.

#### Adverse events and their treatments

**Figure 3:**
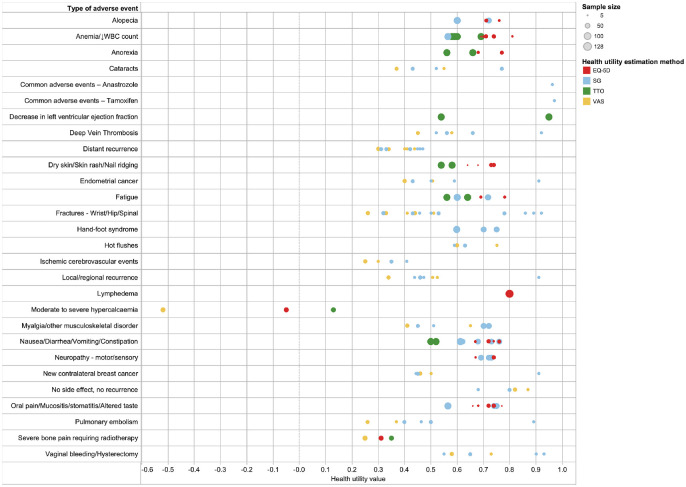
Health utility values for adverse effects of breast cancer treatment interventions.

A total of 10 studies^21,22,25,28,47,51,57,75,76,90^ reported on a range of breast cancer treatment–related adverse events (Figure 3). Lower utilities (<0.5) were found to be associated with fractures, severe bone pain, local or distant recurrence that may or may not require treatment(s), lymphedema, pulmonary embolism, deep vein thrombosis, ischemic cerebrovascular events, endometrial or contralateral breast cancer, and cataracts. The utilities for adverse event–related health states were predominantly estimated using the VAS or SG. VAS values were found to be lower as compared with SG. For health states in which the EQ-5D and TTO were used, utilities estimated using TTO tended to be lower compared with utilities estimated using EQ-5D.

### Breast Cancer Stage-Specific Utilities

**Figure 4:**
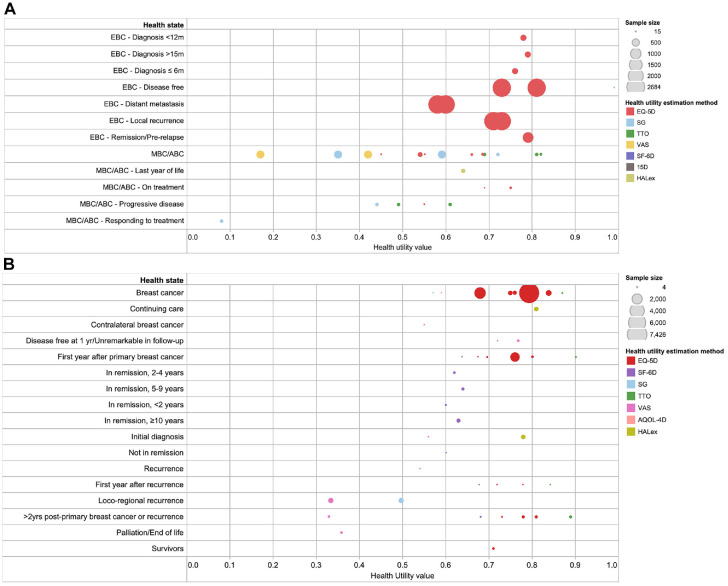
Health utility values in breast cancer, by stage of breast cancer. (a) Early and advanced-stage breast cancer. (b) Nonspecific breast cancer.

A total of 5^12,48,51,87,99^ and 13^9,18,24,29,47,48,53,72,75,82,91,97,101^ studies assessed the utilities for early breast cancer and advanced/metastatic breast cancer–related health states, respectively. As seen in Figure 4a, most of the studies for early breast cancer derived utilities using the EQ-5D. One study with large sample size^99^ (>1000) consistently found the early breast cancer health states to be between 0.58 and 0.81. The health utilities for advanced breast cancer states were mainly measured using direct methods (SG, TTO, and VAS). The utilities for local recurrence were found to be lower than for early breast cancer without recurrence but higher than for advanced or metastatic disease.

Figure 4b shows the utilities for when the stage of breast cancer was not specified (*n* = 20)^11,15,16,19,24,29,30,49,50,53,55,57,64,66,68,69,88,93,97,100^. Several studies with small sample sizes found that the utilities for participants in mid- to long-term remission were lower as compared with locoregional recurrence. The reported utilities from the initial diagnosis of breast cancer to 2 years following primary or recurrent breast cancer were lower as compared with longer term (2 years or more) follow-up.

### Meta-regression

The results of the meta-regression analysis are shown Table 2. For the regression model concerning health utilities by intervention, we found that compared with screening, invasive diagnostic procedures, local therapies, and systemic breast cancer therapies had lower utilities, with the lowest value being in patients undergoing chemotherapy followed by surgery and radiation (*R*^2^ = 0.57). For breast cancer stage, health utilities for advanced or metastatic breast cancer states were lower compared with early breast cancer and when breast cancer stage was not specified (*R*^2^ = 0.37); however, the differences were not statistically significant. For both models, utilities elicited using the VAS were found to be lower compared with the EQ-5D, whereas SG- and TTO-derived utilities were found to be higher than EQ-5D. Lastly, utilities elicited from the general population were found to be lower than from patients.

**Table 2:** Results from Meta-regression Analyses

Variable	Model 1^a^		Model 2^b^	
	Coefficient	*P* Value	Coefficient	*P* Value
Intervention				
Reference: screening				
Noninvasive diagnostic	0.00 (0.00)	0.78	—	—
Invasive diagnostic	–0.06 (0.06)	0.30	—	—
Surgery	–0.15 (0.03)	<0.001*	—	—
Radiation	–0.17 (0.04)	<0.001*	—	—
Surgery and radiation	–0.24 (0.04)	<0.001*	—	—
Chemotherapy	–0.19 (0.04)	<0.001*	—	—
Endocrine therapy	–0.12 (0.03)	<0.001*	—	—
Breast cancer stage				
Reference: early				
Advanced/metastatic	—	—	–0.11 (0.08)	0.18
Non-specific	—	—	–0.02 (0.08)	0.76
Study sample size	0.00 (0.00)	0.53	0.00 (0.00)	0.82
Valuation method				
Reference: EQ-5D				
SG	0.09 (0.03)	<0.01*	0.14 (0.08)	0.09
TTO	0.06 (0.03)	0.08	0.17 (0.06)	<0.05*
VAS	–0.04 (0.02)	<0.05*	–0.03 (0.08)	0.69
Other	–0.01 (0.06)	0.84	–0.01 (0.05)	0.86
Respondents				
Reference: Patients				
Public	–0.04 (0.03)	0.13	–0.26 (0.07)	< 0.01*
Constant	0.92 (0.04)	< 0.001*	0.80 (0.08)	< 0.001*

a By the intervention in model 1: observations: *N* of health utilities in the model = 88; *R*^2^ = 0.57. Studies included in model 1: reference number (number of utilities contributed by the study): 17(2), 21(6), 23(4), 24(14), 26(3), 27(3), 46(4), 48(3), 51(2), 52(1), 55(6), 56(3), 57(1), 60(2), 62(2), 63(1), 64(6), 70(4), 78(4), 79(1), 80(1), 84(3), 87(3), 89(1), 92(2), 94(1), 97(4).

b By breast cancer stage in model 2: observations: *N* of health utilities in the model = 52; *R*^2^ = 0.37. Studies included in model 2: reference number (number of utilities contributed by the study): 9(2), 16(1),18(1),19(1), 24(8), 29(8), 30(3), 48(2), 49(1), 51(1), 53(3), 55(3), 57(1), 64(2), 68(1), 72(1),75(2), 82(1), 88(1), 91(1), 97(4), 99(1), 100(1), 101(2).

* Significant *P* value.

## Discussion

There has been a continued interest in the measurement of health utilities for breast cancer since 2005. This systematic literature review identified the full range of published health utility values relevant to breast cancer from diagnostic or screening, local and systemic therapies, allied health or complementary medicine–related interventions, and treatment-related adverse events. Only 1 of the 79 identified studies in the review explicitly estimated the utility values for Indigenous women with breast cancer,^11^ which is concerning because of the higher incidence and mortality rates of cancer in this population.^31^

We found that the utility values for women undergoing screening and noninvasive diagnostic interventions were equivalent to being in full health. In addition, women with false-positive results on screening mammography and women with confirmed positive results on diagnostic mammography were similar in terms of their health utility values. This observation is supported by a recently published systematic review of 27 studies reporting on the disutilities associated with cancer-screening programs,^32^ which concluded that cancer-screening programs resulted in low disutilities and that diagnostic and treatment programs are associated with low to moderate disutilities. This finding suggests that women experience low health utilities even with suspected breast cancer diagnosis. Subsequently, women should be promptly evaluated for psychological well-being and offered appropriate support and resources to cope with the diagnosis from the outset.

For local interventions, we found that the utility values for breast cancer surgery (i.e., breast-conserving surgery or mastectomy) were lower compared with women undergoing breast reconstruction after cancer surgery. This finding is corroborated by the evidence to date that suggests that undergoing breast reconstructive surgery significantly improves HRQOL and satisfaction with breasts.^33–35^ Health utility values were most assessed for chemotherapy-related health states. The high number of studies reporting on chemotherapy was conceivably due to the rapid turnover of research on new chemotherapeutic agents and possible drug combinations requiring health utility–relevant evidence prior to market entry. This may also have influenced the dominance of generic preference-based measures in this category.

Substantial variation was noted between the different chemotherapy regimens; however, 2 trends were noted. First, patients who were on chemotherapy reported lower values as they progressed in their treatment, such that the values for the last treatment session were lower as compared with the first session. Second, utilities for patients who were on chemotherapy were lower compared with patients who had completed chemotherapy, and utilities after chemotherapy continued to increase. This has important implications when designing cost-effectiveness analyses, as the time frame during which the costs and the benefits accrued may have been different. We did not identify studies estimating utility values for patients on targeted therapies and identified few studies reporting on the utility values for specific HRT drugs. This lack of utility values in the HRT or targeted therapy literature was unforeseen because of the higher uptake of these interventions in breast cancer in recent years.

In terms of breast cancer stage, predictable patterns were observed. We found that the utilities for early-stage breast cancer states were higher compared with advanced or metastatic stages. This finding is similar to Peasgood et al.,^8^ who reported higher values for early-stage breast cancer without recurrence and a sharp decline in progressive advanced or metastatic stage breast cancer health states. An interesting finding was that patients who were in remission (<2 y to ≥10 y) had lower utility values, which indicates the long-term impacts of breast cancer and its associate treatments on HRQOL. It also demonstrates the need to look beyond the treatment phase and assess the costs and HRQOL in patients in the survivor phase.

With respect to the methods used to assess utilities in the breast cancer population, we found that compared with EQ-5D–derived values, utilities elicited using the VAS were lower and SG and TTO were higher. Choice-based methods such as SG and TTO take into account the risk attitude or the tradeoffs in eliciting health state preferences, but only SG incorporates risk preferences. The VAS method is relatively easier but may be prone to response spreading bias^36^ and end-state aversion bias.^37^ Alternatively, indirect methods are inexpensive, easy to implement, and less cognitively burdensome compared with direct application of elicitation tasks and therefore are recommended by some health technology assessment (HTA) agencies. We also found that patients reported higher utility values compared with members of the general public, a finding that is corroborated in the literature. This discrepancy in utility values by population may be due to the hypothetical scenario presented to the general public, adaption to the health state by patients, or potential response shift.^38^ Notably, in the meta-regression analyses, the differential between the patient and public in utility values was found to be much smaller by intervention as compared with stage of breast cancer. This difference may be due to the direct and indirect experiences of the general population for the intervention and breast cancer stage–related health states. The general population may be more familiar with the experience of undergoing oncology treatments. For example, nausea and hair loss are well known side effects of chemotherapy and are often used to typify the image of someone with cancer in the media and other cancer-related reports. In contrast, the general population may not be as aware of the diagnostic, treatment experience, and quality-of-life implications of different stages of cancer. This may cause the general population to report lower utilities compared with patients. Altogether, the context, method, and population used to elicit utility values are important considerations when selecting health utilities for cost-effectiveness analyses.

### Study Limitations

Our study included publications between 2005 and August 2017 in the English language. While our search was comprehensive and designed with the help of a medical librarian, we identified many irrelevant articles, indicating that the search was highly sensitive but lacked precision. More recently, Arber et al.^39,40^ published search filters for Ovid MEDLINE for studies reporting health utility values that maximize sensitivity, balance sensitivity and precision, and maximize precision. The latter 2 strategies may be considered in the future to reduce the number of irrelevant articles. The authors of the publications were not contacted for further clarification or missing data, which might have resulted in the exclusion of relevant studies. Further, we found an increasing trend in the number of studies reporting health utility values from 2012 onward. Our review is limited to studies published until 2017 and does not include recent literature. Reviews of this nature are time-consuming, especially in breast oncology because of the number of publications annually on this topic. Hence, it will be important to update the review findings on an ongoing basis. An important limitation of this review is that we were unable to conduct quality assessment of the included studies. This was due to the lack of such an appraisal tool. To that effect, the senior author is leading a Health Utility Book (HUB) project. Once concluded, the HUB will consist of a registry of published health utility values and a quality assessment tool for health utility studies.^41,42^ We believe that the HUB project will fill an important gap in the literature and improve the credibility of health utility studies in the HTA submission process. Finally, for the meta-regression analysis by breast cancer stage, the results should be interpreted with caution, as the model included only a handful of studies for early-stage breast cancer (*n* = 3) as compared with 18 and 33 studies in advanced and nonspecific breast cancer, respectively. In addition, the purpose of the meta-regression analysis was to evaluate the influence of study characteristics on the utility values and not to generate a utility prediction model. Subsequently, the regression parameters should not be used to generate predicted utility weights.

### Implications: Selecting Health Utility Values for Use in Cost-Effectiveness Models

Given the methodological variation among health utility studies, it is a challenging task to choose from multiple published health utilities for cost-effectiveness models. Recent ISPOR Good Research Practices Task Force Reports^43,44^ recommend evaluating the “fit for purpose” of the health utilities for a cost-effectiveness model by taking into account 1) the population used to elicit utility values (general public versus patient and or caregivers), 2) the population’s sociodemographic (e.g., age, educational level) characteristics, 3) clinical (e.g., stage of breast cancer, HER2 status, menopausal status, comorbidities) and treatment (e.g., active treatment versus survivorship, treatment-related adverse events, or acute clinical events) characteristics, 4) description of the health state (individual health state versus aggregate as a function of the clinical status), and 5) utility study design and setting (e.g. country, sample size, mode of administration). We believe the catalog of the breast cancer–related health utilities provided in our study could be useful for future cost-effectiveness models, whereby the authors will be able to weigh the aforementioned characteristics and ascertain the value(s) to use for their analysis.

## Conclusion

This study provides a catalog of the published health utility values related to breast cancer. We found that even though a variety of utility estimation methods have been used in the breast cancer literature, the EQ-5D is most commonly used. A higher proportion of studies identified in the review measured health utilities for interventions, more specifically for chemotherapy and treatment-related adverse events. Further, substantial variation was noted in the utility values among studies reporting on the same health state. This variation was larger for systemic interventions and treatment-related adverse events than for screening-, diagnostic-, local-, or allied health and complementary medicine–related interventions. The utilities for early breast cancer health states were higher compared with the advanced or metastatic breast cancer health states.

Another important contribution of this systematic literature review is understanding the source of variation in utility values by method, population, and type of health state (i.e., intervention or breast cancer stage). The catalog may be useful for future economic evaluations; however, a word of caution is in order due to the heterogeneity of published utilities.

## Supplemental Material

sj-docx-1-mdm-10.1177_0272989X211065471 – Supplemental material for A Systematic Literature Review of Health Utility Values in Breast Cancer Click here for additional data file. Supplemental material, sj-docx-1-mdm-10.1177_0272989X211065471 for A Systematic Literature Review of Health Utility Values in Breast Cancer by Manraj N. Kaur, Jiajun Yan, Anne F. Klassen, Justin P. David, Dilshan Pieris, Manraj Sharma, Louise Bordeleau and Feng Xie in Medical Decision Making
