# Supplementary material for: A Systematic Literature Review of Health Utility Values in Breast Cancer
Source: Med Decis Making. 2022 Jan 18;42(5):704–19. doi: 10.1177/0272989X211065471 (PMC9189726; doi:10.1177/0272989X211065471)
Supplement: sj-docx-1-mdm-10.1177_0272989X211065471 – Supplemental material for A Systematic Literature Review of Health Utility Values in Breast Cancer [file sj-docx-1-mdm-10.1177_0272989X211065471.docx]

**Supplementary Material**

**Table 1: MEDLINE search strategy for the systematic review of literature of published health utility values in breast cancer**

| 1. exp breast neoplasms/ or exp mammary neoplasms/ |
| --- |
| 1. (breast$ adj5 (neoplas$ or cancer$ or tumo?r$ or carcinoma$ or adenocarcinoma$ or sarcoma$ or dcis or ductal or infiltrat$ or intraductal$ or lobular or medullary)).tw. |
| 1. (mammar$ adj5 (neoplas$ or cancer$ or tumo?r$ or carcinoma$ or adenocarcinoma$ or sarcoma$ or dcis or ductal or infiltrat$ or intraductal$ or lobular or medullary)).tw. |
| 1. or/1-3 |
| 1. (euroqol or euro QOL or eq5d or eq 5D).tw. |
| 1. (sf6D or sf 6D or short form 6D or shortform 6D or sf six D or sfsixD or shortform six D or short form sixD or sf6d or 6d or 6d or 6 dimension).tw. |
| 1. Health Utilities Index.mp. or health utilit$.tw. or hui.tw. |
| 1. (Utility Based Questionnaire-Cancer or UBQC or UBQC or UBQ C).tw. |
| 1. (Qaly$ or quality adjusted life year$).tw. |
| 1. (Hye$ or health$ year$ equivalent$).tw. |
| 1. (quality of well being or quality of wellbeing).tw |
| 1. (qald$ or qale$ or qtimes$ or (quality adjusted life day$ or quality adjusted life expectanc$ or quality adjusted survival$)).tw. |
| 1. Standard gamble$.tw. |
| 1. (time trade off or time tradeoff or tto).tw. |
| 1. visual analog$ scale$.tw. |
| 1. discrete choice experiment$.tw. |
| 1. (health state$ utilit$ or health state$ value$ or health state$ preference$).tw. |
| 1. (cost* adj2 (effective* or utilit* or benefit* or minimi* or evaluat* or analy*)).tw. |
| 1. or/5-18 |
| 1. 4 and 19 |
| 1. c |
| 1. limit 21 to yr="2005-Current" |

**Table 2: Characteristics of the studies included in the review (N=79)**

| Study (Author last name) | Country of corresponding author | Study design for preference elicitation study | Respondents | Health utility estimation method |
| --- | --- | --- | --- | --- |
| Conner-Spady et al. (2005)ˢ | Canada | Cohort - prospective | Patients | EQ-5D (UK), QOL VAS |
| Gordon et al. (2005)*ˢ | Australia | Cohort - prospective | Patients | SHE |
| Hayman et al. (2005)*ˢ | USA | Cross-sectional | Public (w), Patients | SG |
| Lloyd et al. (2006)* | UK | Cross-sectional | Public | EQ-5D (UK), SG, VAS |
| Milne et al. (2006) | New Zealand | Cross-sectional | Public (w) | EQ-5D (NZ, UK), TTO, VAS |
| Schleinitz et al. (2006)ˢ | USA | Cross-sectional | Public (w) | SG, TTO |
| Shih et al. (2006) | USA | Cross-sectional | Patients | SF-6D |
| Sullivan et al. (2006) | USA | Cross-sectional | Patients | EQ-5D (US) |
| Lidgren et al. (2007) | Sweden | Cross-sectional | Patients | EQ-5D (UK), TTO |
| Mansel et al. (2007)* | UK | Cross-sectional | Patients | SG |
| Prescott et al. (2007)ˢ | UK | RCT | Patients | EQ-5D (UK) |
| Yabroff et al. (2007) | USA | Cross-sectional | Patients | HALex |
| Bernhard et al. (2008)*ˢ | Switzerland | RCT | Patients | SHE, TTO |
| Bonomi et al. (2008) | USA | Cross-sectional | Public (w) | VAS |
| Melnikow et al. (2008)ˢ | USA | Cross-sectional | Public (w) | SG |
| Sherrill et al. (2008)* | USA | RCT | Patients | EQ-5D (UK) |
| Wolowacz et al. (2008)* | UK | Cross-sectional | Patients | EQ-5D^ (UK) |
| Dranitsaris et al. (2009)* | Canada | Cross-sectional | HCP | TTO |
| Fountzilas et al. (2008)* | Greece | RCT | Patients | EQ-5D (Europe) |
| Reed et al. (2009)* | USA | RCT | Patients | HUI 3 |
| Zhou et al. (2009)* | USA | RCT | Patients | EQ-5D (UK) |
| Cheville et al. (2010)* | USA | Cross-sectional | Patients | EQ-5D (US), TTO |
| De Kok et al. (2010)*ˢ | The Netherlands | Cohort - prospective | Patients | EQ-5D (UK) |
| Domeyer et al. (2010) | Greece | Cohort - prospective | Public (w) | EQ-5D (ES) |
| Freedman et al. (2010)* | USA | Cohort - prospective | Patients | EQ-5D (UK) |
| Grann et al. (2010)*ˢ | USA | Cross-sectional | Patients | TTO |
| Haines et al. (2010)*ˢ | Australia | RCT | Patients | EQ-5D (UK) |
| Lux et al. (2010)* | Germany | Cross-sectional | Patients | VAS |
| Kimman et al. (2011)*ˢ | The Netherlands | RCT | Patients | EQ-5D (UK) |
| Matalqah et al. (2011) | Malaysia | Cross-sectional | Public (w), Patients | EQ-5D (UK) |
| Sullivan et al. (2011) | USA | Cross-sectional | Patients | EQ-5D (UK) |
| Shirowa et al. (2011) | Japan | RCT | Patients | EQ-5D (UK) |
| Bastani et al. (2012)ˢ | Iran | Cohort - prospective | Patients | EQ-5D^ |
| Cheng et al. (2012)* | Taiwan | Cohort - retrospective | Patients | SG |
| Oh et al. (2012)* | Korea | Cross-sectional | Patients | EQ-5D (KR) |
| Robertson et al. (2012)*ˢ | Sweden | Cross-sectional | Patients | EQ-5D (UK) |
| Serra et al. (2012)* | USA | Cohort - prospective | Patients | EQ-5D |
| Shih et al. (2012)ˢ | Singapore | Cross-sectional | HCP | SG, VAS |
| Frederix et al. (2013) | UK | Cross-sectional | Public | VAS, TTO |
| Kuchuk et al. (2013)* | Canada | Cross-sectional | Patients | SG |
| Moro-Valdezate et al. (2013)* | Spain | Cohort - Prospective | Patients | EQ-5D (ES) |
| Postma et al. (2013)* | The Netherlands | RCT | Patients | EQ-5D |
| Sinno et al. (2013)* | Canada | Cross-sectional | Public | VAS, TTO, SG |
| Arving et al. (2014)*ˢ | Sweden | RCT | Patients | EQ-5D^ (UK) |
| Farkkila et al. (2014) | Finland | Cross-sectional | Patients | EQ-5D (UK), 15D |
| Humphrey et al. (2014)*ˢ | USA | Cross-sectional | Public (w) | TMI |
| Min et al. (2014)ˢ | Korea | Cross-sectional | Patients | EQ-5D |
| Moro-Valdezate et al. (2014)* | Spain | Cohort - prospective | Patients | EQ-5D (ES) |
| Sinno et al. (2014)* | USA | Cross-sectional | Public | VAS, TTO, SG |
| Songtish et al. (2014)ˢ | Thailand | Cross-sectional | Public (w) | SG |
| Tan et al. (2014)ˢ | Singapore | Cross-sectional | Patients | SG, VAS |
| Timmers et al. (2014)ˢ | The Netherlands | RCT | Public (w) | EQ-5D (NL) |
| Tosteson at al. (2014)ˢ | Lebanon | Cross-sectional | Public (w) | EQ-5D (US) |
| Dranitsaris et al. (2015) | Canada | Cross-sectional | Patients | TTO |
| Eyles et al. (2015)*ˢ | England | Cohort - prospective | Patients | EQ-5D |
| Hall et al. (2015)*ˢ | UK | Cohort - prospective | Patients | EQ-5D (UK) |
| Kimman et al. (2015) | Australia | Cross-sectional | Patients | EQ-5D (TH) |
| Swan et al. (2015)*ˢ | USA | Cross-sectional | Public (w) | TMI |
| Tachi et al. (2015) | Japan | Cohort - prospective | Patients | EQ-5D (JP) |
| Brown et al. (2016)ˢ | USA | Cross-sectional | Public (w) | EQ-5D^ (US) |
| Garvey et al. (2016)ˢ | Australia | Cohort - prospective | Patients | AQOL-4D |
| Pickard et al. (2016) | USA | Cohort - retrospective | Patients | EQ-5D (US) |
| Shirowa et al. (2016)* | Japan | RCT | Patients | EQ-5D (JP) |
| Trogdon et al. (2016)ˢ | USA | Cross-sectional | Public, Patients | EQ-5D^ (US) |
| Wang et al. (2016) | USA | Cross-sectional | Public, Patients | SF-6D^ |
| Yagata et al. (2016)*ˢ | Japan | RCT | Patients | EQ-5D (JP) |
| Yousefi et al. (2016)ˢ | Iran | Cross-sectional | Patients | EQ-5D (UK), SF-6D |
| Ali et al. (2017)ˢ | USA | Cohort - retrospective | Patients | SF-6D, VR-6D^ |
| Enblom et al. (2017)*ˢ | Sweden | Cross-sectional | Patients | EQ-5D (UK) |
| Gordon et al. (2017)*ˢ | Australia | RCT | Patients | EQ-5D (AU) |
| Kim et al. (2017)ˢ | South Korea | Cross-sectional | Public | SG, VAS |
| Knuttel et al. (2017) | The Netherlands | Cross-sectional | Public (w), Patients | EQ-5Dᶿ (UK), TTO |
| Liu et al. (2017)ˢ | China | Cross-sectional | Patients | EQ-5Dᶿ (UK, CHN, JP, KR) |
| May et al. (2017)ˢ | The Netherlands | RCT | Patients | EQ-5D (NL) |
| Naik et al. (2017)ˢ | Canada | Cross-sectional | Patients | EQ-5D (UK, CA, US) |
| Seferina et al. (2017)* | The Netherlands | Cross-sectional | Patients | EQ-5D (UK) |
| The ACTION Study Group (2017) | Australia, Netherlands | Cohort - prospective | Patients | EQ-5D (TH) |
| van Kampen et al. (2017)*ˢ | The Netherlands | Cross-sectional | Patients | EQ-5D (UK) |
| Wallwiener et al. (2017) | Germany | Cross-sectional | Patients | EQ-5Dᶿ |

ˢReceived funding from not-for-profit sources; *Study published in Oncology journal; ᶿ5-L version; ^mapping study; AU, Australia; CA, Canada; CHN, China; JP, Japan; KR, Korea; NL, the Netherlands; NZ, New Zealand; ES, Spain; TH, Thailand; UK, United Kingdom; US, United States of America; (w), women only; RCT, Randomized Controlled Trial; HCP, Health Care Provider; SHE, Subjective Health Estimation; SG, Standard Gamble; VAS, Visual Analogue Scale; TMI, Testing Morbidities Index; TTO, Time Trade Off; SF-6D, Short-form 6D; HALeX, Health and Activities Limitation Index

**Table 3: Health utility values by breast cancer intervention**

1. **Screening-related health states**

| Health state | | Respondents | Sample size | Age in years (mean, mean ± standard deviation is provided when available) | Health utility estimation method | Health Utility value (mean, mean ± standard deviation when available | Study |
| --- | --- | --- | --- | --- | --- | --- | --- |
| Screening mammography |  | Public (w) (10% with history of BC) | 131 | 65% <64 | VAS | 0.80 ± 0.14 | Bonomi et al. (2008) |
|  |  | Public (w) – BRCA+ | 83 | 67.5% - 35-60 | TTO | 1.00 ± 0.00 | Grann et al. (2010) |
|  |  | Public | 60 | 43.1% - 18-34 | TTO | 0.97 ± 0.11 | Grann et al. (2010) |
| Screening mammography – negative^1^ |  | Public (w) (10% with history of BC) | 131 | 65% <64 | VAS | 0.89 ± 0.10 | Bonomi et al. (2008) |
|  |  | Public (w) | 531 | 48.7% - 50-64 | EQ-5D | 0.90 ± 0.13 | Tosteson et al. (2014) |
| Screening mammography - false positive^1^ |  | Public (w) (10% with history of BC) | 131 | 65% <64 | VAS | 0.49 ± 0.21 | Bonomi et al. (2008) |
|  | Unresolved | Public (w) | 259 | 44% <50 | EQ-5D | 0.90 ± 0.13 | Tosteson et al. (2014) |
|  | Resolved | Public (w) | 235 | 44% <50 | EQ-5D | 0.91 ±0.13 | Tosteson et al. (2014) |
| Diagnostic follow-up* | With general practitioner – pre-workup | Public (w) | 91 | 57 | EQ-5D | 0.89 | Timmers et al. (2014) |
|  | With general practitioner – post-workup | Public (w) | 91 | 57 | EQ-5D | 0.91 | Timmers et al. (2014) |
|  | With radiologist – pre-workup | Public (w) | 245 | 57 | EQ-5D | 0.85 | Timmers et al. (2014) |
|  | With radiologist – post-workup | Public (w) | 245 | 57 | EQ-5D | 0.89 | Timmers et al. (2014) |

^1^Baseline values are shown; values for one year after baseline are not shown as similar to baseline values; *not a diagnostic intervention but a follow-up strategy; (w), women only; BI-RADS, Breast Imaging Reporting and Data System; BRCA, Breast Cancer gene; EQ-5D, Euro-QOL 5D; VAS, Visual Analogue Scale; TMI, Testing Morbidity Index; TTO, Time trade-off

1. Non-invasive diagnosis-related health states

| Health state | | Respondents | Sample size | Age in years (mean, mean ± standard deviation is provided when available) | Health utility estimation method | Health Utility value (mean, mean ± standard deviation when available | Study |
| --- | --- | --- | --- | --- | --- | --- | --- |
| Diagnostic mammography |  | Public (w) (10% with history of BC) | 131 | 65% <64 | VAS | 0.55 ± 0.20 | Bonomi et al. (2008) |
| Diagnostic mammography - true positive |  | Public (w) (10% with history of BC) | 131 | 65% <64 | VAS | 0.46 ± 0.21 | Bonomi et al. (2008) |
| Diagnostic mammography - false positive |  | Public (w) (10% with history of BC) | 131 | 65% <64 | VAS | 0.81 ± 0.15 | Bonomi et al. (2008) |
| MRI |  | Public (w) – BRCA+ | 83 | 67.5% - 35-60 | TTO | 1.00 ± 0.01 | Grann et al. (2010) |
|  |  | Public | 60 | 43.1% - 18-34 | TTO | 0.96 ± 0.10 | Grann et al. (2010) |

1. Invasive diagnostic procedure-related health states

| Health state | | Respondents | Sample size | Age in years (mean, mean ± standard deviation is provided when available) | Health utility estimation method | Health Utility value (mean, mean ± standard deviation when available | Study |
| --- | --- | --- | --- | --- | --- | --- | --- |
| Biopsy - Vacuum-assisted | Pre-biopsy | Public (w) - non-palpable lesions (BI-RADS 3/4) | 102 | 51.3 ± 8.8 | EQ-5D | 0.73 ± 0.22 | Domeyer et al. (2010) |
|  | Pre-biopsy | Public (w) - non-palpable lesions (BI-RADS 3/4) | 102 | 51.3 ± 8.8 | EQ-5D VAS | 0.83 ± 0.08 | Domeyer et al. (2010) |
|  | Post-Early | Public (w) - non-palpable lesions (BI-RADS 3/4) | 102 | 51.3 ± 8.8 | EQ-5D | 0.79 ± 0.21 | Domeyer et al. (2010) |
|  | Post-Early | Public (w) - non-palpable lesions (BI-RADS 3/4) | 102 | 51.3 ± 8.8 | EQ-5D VAS | 0.85 ± 0.06 | Domeyer et al. (2010) |
|  | Post-Late | Public (w) - non-palpable lesions (BI-RADS 3/4) | 102 | 51.3 ± 8.8 | EQ-5D | 0.77 ± 0.23 | Domeyer et al. (2010) |
|  | Post-Late | Public (w) - non-palpable lesions (BI-RADS 3/4) | 102 | 51.3 ± 8.8 | EQ-5D VAS | 0.85 ± 0.09 | Domeyer et al. (2010) |
| Biopsy - Percutaneous |  | Public (w) | 188 | 51.4 | TMI | 0.84 ± 0.06 | Humphrey et al. (2014) |
| Biopsy - Core needle |  | Public (w) | 100 | 51 | TMI | 0.84 ± 0.07 | Swan et al. (2015) |
| Radio-occult lesion localization | Baseline | Patients | 162 | 60.5 ± 7.7 | EQ-5D | 0.86 ± 0.16 | Postma et al. (2013) |
|  | Baseline | Patients | 162 | 60.5 ± 7.7 | EQ-5D VAS | 0.77 ± 0.12 | Postma et al. (2013) |
|  | 12-months | Patients | 151 | 60.5 ± 7.7 | EQ-5D | 0.84 ± 0.19 | Postma et al. (2013) |
|  | 12-months | Patients | 151 | 60.5 ± 7.7 | EQ-5D VAS | 0.72 ± 0.13 | Postma et al. (2013) |
| Wire-guided localization | Baseline | Patients | 152 | 61.1 ± 9.7 | EQ-5D | 0.83 ± 0.18 | Postma et al. (2013) |
|  | Baseline | Patients | 152 | 61.1 ± 9.7 | EQ-5D VAS | 0.75 ± 0.15 | Postma et al. (2013) |
|  | 12-months | Patients | 141 | 61.1 ± 9.7 | EQ-5D | 0.80 ± 0.20 | Postma et al. (2013) |
|  | 12-months | Patients | 141 | 61.1 ± 9.7 | EQ-5D VAS | 0.72 ± 0.14 | Postma et al. (2013) |

1. **Local therapy**
   1. **Surgery**

| Health state | | Respondents | Sample size | Age in years (mean, mean ± standard deviation is provided when available) | Health utility estimation method | Health Utility value (mean, mean ± standard deviation when available | Study |
| --- | --- | --- | --- | --- | --- | --- | --- |
| Oncological surgery | 1-year | Patients | 364 | 59 ± 13 | EQ-5D | 0.83^**^ | Moro-Valdezate et al.(2014) |
|  | Within ≤ 6m of diagnosis | Patients | 297 | 57 ± 10.7 | EQ-5D | 0.76 | Hall et al. (2015) |
|  | Within <12m of diagnosis | Patients | 297 | 57 ± 10.7 | EQ-5D | 0.78 | Hall et al. (2015) |
|  | Within >15m of diagnosis | Patients | 297 | 57 ± 10.7 | EQ-5D | 0.79 | Hall et al. (2015) |
| Breast conserving surgery |  | Patients | 146 | 76.64 ± 7.09 | VR-6D | 0.68 ± 0.13 | Ali et al. (2017) |
|  |  | Public (w) (10% with history of BC) | 131 | 65% <64 | VAS | 0.53 ± 0.21 | Bonomi et al. (2008) |
|  |  | Patients | 120 | 61 ± 10 | SG | 0.91 ± 0.15 | Hayman et al (2005) |
|  |  | Public (w) | 210 | 50 ± 14 | SG | 0.90 ± 0.15 | Hayman et al. (2005) |
|  | 1-year | Patients | 227 | 59 ± 13 | EQ-5D | 0.88^**^ | Moro-Valdezate et al.(2014) |
|  | EBC | Public (w) | 110 | 26 – 60 | SG | 0.76 (SE=0.04) | Songtish et al.(2014) |
|  | EBC, with lymphedema | Public (w) | 110 | 26 – 60 | SG | 0.59 (SE=0.04) | Songtish et al.(2014) |
| Mastectomy |  | Public (w) (10% with history of BC) | 131 | 65% <64 | VAS | 0.48 ± 0.22 | Bonomi et al. (2008) |
|  |  | Public | 509 | 45.7 ± 14.1 | VAS | 0.67 ± 0.20 | Kim et al. (2017) |
|  |  | Public | 509 | 45.7 ± 14.1 | SG | 0.79 ± 0.27 | Kim et al. (2017) |
|  | 1-year | Patients | 137 | 59 ± 13 | EQ-5D | 0.80^**^ | Moro-Valdezate et al.(2013) |
| Mastectomy – unilateral |  | Public | 140 | 23.6 ± 7.0 | VAS | 0.75 ± 0.17 | Sinno et al. (2014) |
|  |  | Public | 141 | 23.6 ± 7.0 | TTO | 0.87 ± 0.14 | Sinno et al. (2014) |
|  |  | Public | 142 | 23.6 ± 7.0 | SG | 0.86 ± 0.18 | Sinno et al. (2014) |
| Mastectomy – bilateral |  | Public | 120 | 24.8 ± 8.4 | VAS | 0.70 ± 0.18 | Sinno et al. (2013) |
|  |  | Public | 120 | 24.8 ± 8.4 | TTO | 0.85 ± 0.16 | Sinno et al. (2013) |
|  |  | Public | 120 | 24.8 ± 8.4 | SG | 0.86 ± 0.17 | Sinno et al. (2013) |
| Mastectomy with sentinel lymph node biopsy |  | Patients | 71 | 60.1 ± 9.4 | VAS | 0.80 (0.70-0.90) ^**^ | Knuttel et al. (2017) |
|  |  | Patients | 71 | 60.1 ± 9.4 | TTO | 0.90 (0.80-0.95) ^**^ | Knuttel et al. (2017) |
|  |  | Public | 50 | 56.1 ± 9.4 | VAS | 0.70 (0.60-0.80) ^**^ | Knuttel et al. (2017) |
|  |  | Public | 50 | 56.1 ± 9.4 | TTO | 0.80 (0.80-0.95) ^**^ | Knuttel et al. (2017) |
| Mastectomy after breast conserving surgery | Recurrence- DCIS | Patients | 120 | 61 ± 10 | SG | 0.87 ± 0.21 | Hayman et al. (2005) |
|  |  | Public (w) | 210 | 50 ± 14 | SG | 0.89 ± 0.15 | Hayman et al. (2005) |
|  | Recurrence – IBC | Patients | 120 | 61 ± 10 | SG | 0.75 ± 0.29 | Hayman et al. (2005) |
|  |  | Public (w) | 210 | 50 ± 14 | SG | 0.84 ± 0.18 | Hayman et al. (2005) |
| Mastectomy followed by reconstruction | Stage 0 | Public | 509 | 45.7 ± 14.1 | SG | 0.80 ± 0.26 | Kim et al. (2017) |
|  |  | Public | 509 | 45.7 ± 14.1 | VAS | 0.68 ± 0.20 | Kim et al. (2017) |
| Mastectomy with sentinel lymph node biopsy and implant-based reconstruction |  | Patients | 71 | 60.1 ± 9.4 | VAS | 0.70 (0.60-0.85) ^**^ | Knuttel et al. (2017) |
|  |  | Patients | 71 | 60.1 ± 9.4 | TTO | 0.90 (0.75-0.95) ^**^ | Knuttel et al. (2017) |
|  |  | Public | 50 | 56.1 ± 9.4 | VAS | 0.73 (0.64-0.85) ^**^ | Knuttel et al. (2017) |
|  |  | Public | 50 | 56.1 ± 9.4 | TTO | 0.85 (0.70-0.95) ^**^ | Knuttel et al. (2017) |
| Immediate implant-based reconstruction |  | Patients | 164 | 50 | EQ-5D | 0.83 | Robertson et al. (2012) |
| Mastectomy – Prophylactic |  | Public (w) - BRCA+ | 83 | 67.5% - 35-60 | TTO | 0.88 ± 0.22 | Grann et al. (2010) |
|  |  | Public (w) | 60 | 40% - 35-50 | TTO | 0.88 ± 0.17 | Grann et al. (2010) |

**median values; BC, Breast Cancer; (w), women only; BRCA, BReast CAncer gene; EBC, Early breast cancer; EQ-5D, Euro-QOL 5D; VAS, Visual Analogue Scale; TTO, Time Trade Off; SG, Standard Gamble; VR-6D, Veterans RAND-6D

- 1. **Surgery and radiation**

| Health state | | Respondents | Sample size | Age in years (mean, mean ± standard deviation is provided when available) | Health utility estimation method | Health Utility value (mean, mean ± standard deviation when available | Study |
| --- | --- | --- | --- | --- | --- | --- | --- |
| Breast conserving surgery and radiation | Stage 0 | Public | 509 | 45.7 ± 14.1 | SG | 0.78 ± 0.26 | Kim et al. (2017) |
|  |  | Public | 509 | 45.7 ± 14.1 | VAS | 0.66 ± 0.20 | Kim et al. (2017) |
|  |  | Patients | 472 | 72.53 ± 5.41 | VR-6D | 0.70 ± 0.12 | Ali et al. (2017) |
|  | Stage 0-II, 1 year | Patients | 482 | 57% - 45-64 | EQ-5D | 0.87 | Freedman et al (2010) |
|  | Stage 0-II, 5 years | Patients | 171 | 57% - 45-64 | EQ-5D | 0.89 | Freedman et al (2010) |
|  | Stage 0-II, 10 years | Patients | 64 | 57% - 45-64 | EQ-5D | 0.90 | Freedman et al (2010) |
|  | Stage 0-II, 15 years | Patients | 21 | 57% - 45-64 | EQ-5D | 0.90 | Freedman et al (2010) |
| Breast conserving surgery with sentinel lymph node biopsy and whole breast radiation |  | Patients | 71 | 60.1 ± 9.4 | VAS | 0.90 (0.8-0.90)** | Knuttel et al.(2017) |
|  |  | Patients | 71 | 60.1 ± 9.4 | TTO | 0.95 (0.90–1.00) ** | Knuttel et al.(2017) |
|  |  | Public | 50 | 56.1 ± 9.4 | VAS | 0.75 (0.65-0.90) ** | Knuttel et al.(2017) |
|  |  | Public | 50 | 56.1 ± 9.4 | TTO | 0.83 (0.80-0.95) ** | Knuttel et al.(2017) |
| Breast conserving surgery or mastectomy, radiation, and/or chemotherapy | Stages I, II | Public | 509 | 45.7 ± 14.1 | SG | 0.73 ± 0.26 | Kim et al. (2017) |
|  |  | Public | 509 | 45.7 ± 14.1 | VAS | 0.58 ± 0.20 | Kim et al. (2017) |
| Mastectomy and radiation | Stages IIIA, IIIB | Public | 509 | 45.7 ± 14.1 | SG | 0.61 ± 0.26 | Kim et al. (2017) |
|  |  | Public | 509 | 45.7 ± 14.1 | VAS | 0.44 ± 0.18 | Kim et al. (2017) |
| Breast conserving surgery followed by mastectomy and radiation | Recurrence- DCIS | Patients | 120 | 61 ± 10 | SG | 0.89 ± 0.16 | Hayman et al. (2005) |
|  |  | Public (w) | 210 | 50 ± 14 | SG | 0.88 ± 0.16 | Hayman et al. (2005) |
|  | Recurrence – IBC | Patients | 120 | 61 ± 10 | SG | 0.80 ± 0.27 | Hayman et al. (2005) |
|  |  | Public (w) | 210 | 50 ± 14 | SG | 0.81 ± 0.19 | Hayman et al. (2005) |
| Repeat breast conserving surgery and radiation | Recurrence- DCIS | Patients | 120 | 61 ± 10 | SG | 0.89 ± 0.19 | Hayman et al. (2005) |
|  |  | Public (w) | 210 | 50 ± 14 | SG | 0.90 ± 0.15 | Hayman et al. (2005) |
|  | Recurrence – IBC | Patients | 120 | 61 ± 10 | SG | 0.79 ± 0.26 | Hayman et al. (2005) |
|  |  | Public (w) | 210 | 50 ± 14 | SG | 0.81 ± 0.19 | Hayman et al. (2005) |

**median values; DCIS, Ductal Carcinoma In-situ; IBC, Invasive Breast Cancer; (w), women only; VAS, Visual Analogue Scale; TTO, Time Trade Off; SG, Standard Gamble; VR-6D, Veterans RAND-6D

- 1. **Radiation therapy**

| Health state | | Respondents | Sample size | Age in years (mean, mean ± standard deviation is provided when available) | Health utility estimation method | Health Utility value (mean, mean ± standard deviation when available | Study |
| --- | --- | --- | --- | --- | --- | --- | --- |
| Radiation |  | Public (10% history of BC) | 131 | 50-79 | VAS | 0.46 **±** 0.23 | Bonomi et al. (2008) |
|  |  | Public (w) | 156 | 46.2% ≥ 50 | TTO | 0.61 | Schleinitz et al. (2006) |
|  | baseline | Patients | 102 | 72.3 **±** 5.0 | EQ-5D | 0.77 | Prescott et al. (2007) |
|  | 3.5m | Patients | 102 | 72.3 **±** 5.0 | EQ-5D | 0.78 | Prescott et al. (2007) |
|  | 9m | Patients | 102 | 72.3 **±** 5.0 | EQ-5D | 0.76 | Prescott et al. (2007) |
|  | 15m | Patients | 102 | 72.3 **±** 5.0 | EQ-5D | 0.74 | Prescott et al. (2007) |
|  | Within ≤ 6m of diagnosis | Patients | 297 | 57 ± 10.7 | EQ-5D | 0.76 | Hall et al. (2015) |
|  | Within <12m of diagnosis | Patients | 297 | 57 ± 10.7 | EQ-5D | 0.77 | Hall et al. (2015) |
|  | Within >15m of diagnosis | Patients | 297 | 57 ± 10.7 | EQ-5D | 0.81 | Hall et al. (2015) |
| No radiation | baseline | Patients | 101 | 72.8 **±** 5.2 | EQ-5D | 0.74 | Prescott et al. (2007) |
|  | 3.5m | Patients | 101 | 72.8 **±** 5.2 | EQ-5D | 0.76 | Prescott et al. (2007) |
|  | 9m | Patients | 101 | 72.8 **±** 5.2 | EQ-5D | 0.72 | Prescott et al. (2007) |
|  | 15m | Patients | 101 | 72.8 **±** 5.2 | EQ-5D | 0.73 | Prescott et al. (2007) |
| Radiofrequency ablation preceded by SLNB and followed by whole breast radiotherapy^^ |  | Patients | 71 | 60.1 **±** 9.4 | VAS | 0.80* | Knuttel et al.(2017) |
|  |  | Patients | 71 | 60.1 **±** 9.4 | TTO | 0.90* | Knuttel et al.(2017) |
|  |  | Public | 50 | 56.1 **±** 9.4 | VAS | 0.78* | Knuttel et al.(2017) |
|  |  | Public | 50 | 56.1 **±** 9.4 | TTO | 0.80* | Knuttel et al.(2017) |
| Magnetic resonance guided high intensity focused ultrasound preceded by SLNB followed by whole breast radiotherapy^^ |  | Patients | 71 | 60.1 **±** 9.4 | VAS | 0.80* | Knuttel et al.(2017) |
|  |  | Patients | 71 | 60.1 **±** 9.4 | TTO | 0.88* | Knuttel et al.(2017) |
|  |  | Public | 50 | 56.1 **±** 9.4 | VAS | 0.80* | Knuttel et al.(2017) |
|  |  | Public | 50 | 56.1 **±** 9.4 | TTO | 0.85* | Knuttel et al.(2017) |
| Ablative tumor radiation (single dose) preceded by SLNB^^ |  | Patients | 71 | 60.1 **±** 9.4 | VAS | 0.77* | Knuttel et al.(2017) |
|  |  | Patients | 71 | 60.1 **±** 9.4 | TTO | 0.85* | Knuttel et al.(2017) |
|  |  | Public | 50 | 56.1 **±** 9.4 | VAS | 0.80* | Knuttel et al.(2017) |
|  |  | Public | 50 | 56.1 **±** 9.4 | TTO | 0.88* | Knuttel et al.(2017) |

^^techniques that use heat (i.e., ultrasound) with or without radiation – included here for classification purposes; *median values; BC, Breast Cancer; (w), women only; EQ-5D, Euro-QOL 5D; VAS, Visual Analogue Scale; TTO, Time Trade Off

1. **Systemic therapy**
   1. **Chemotherapy – drug not specified**

| Health state | | Respondents | Sample size | Age in years (mean, mean ± standard deviation is provided when available) | Health utility estimation method | Health Utility value (mean, mean ± standard deviation when available | Study |
| --- | --- | --- | --- | --- | --- | --- | --- |
| Before chemotherapy |  | Patients | 52 | 44.7 **±** 8.5 | EQ-5D | 0.78 **±** 0.18 | Conner-Spady et al. (2005) |
|  |  | Patients | 52 | 44.7 **±** 8.5 | QOL-VAS | 0.75 **±** 0.04 | Conner-Spady et al. (2005) |
|  |  | Patients | 47 | 59.6 **±** 12.2 | EQ-5D | 0.84 **±** 0.10 | Tachi et al. (2015) |
| Chemotherapy |  | Public (10% with history of BC) | 131 | 50-79 | VAS | 0.40 **±** 0.21 | Bonomi et al. (2008) |
|  | First year post-diagnosis | Patients | 23 | 57 | EQ-5D | 0.62 | Lidgren et al. (2007) |
|  | First year post-diagnosis | Patients | 22 | 57 | TTO | 0.87 | Lidgren et al. (2007) |
|  |  | Patients | 30 | 45 **±** 6 | EQ-5D | 0.92 ± 0.09 | Min et al. (2014) |
|  |  | Public (w) | 156 | 46.2% ≥ 50 | TTO | 0.48 | Schleinitz et al. (2006) |
|  | Within ≤ 6m of diagnosis | Patients | 297 | 57 ± 10.7 | EQ-5D | 0.75 | Hall et al. (2015) |
|  | Within <12m of diagnosis | Patients | 297 | 57 ± 10.7 | EQ-5D | 0.76 | Hall et al. (2015) |
|  | Within >15m of diagnosis | Patients | 297 | 57 ± 10.7 | EQ-5D | 0.81 | Hall et al. (2015) |
| Chemotherapy – recurrence | Loco-regional and/or contralateral recurrence | Patients | 7 | 57 | EQ-5D | 0.77 | Lidgren et al. (2007) |
|  |  | Patients | 5 | 57 | TTO | 0.86 | Lidgren et al. (2007) |
|  | Distant/metastatic | Patients | 26 | 68 | SG | 0.71 **±** 0.25 | Mansel et al. (2007) |
|  |  | Patients | 38 | 57 | EQ-5D | 0.69 | Lidgren et al. (2007) |
|  |  | Patients | 35 | 57 | TTO | 0.78 | Lidgren et al. (2007) |
| After chemotherapy |  | Patients | 47 | 59.6 **±** 12.2 | EQ-5D | 0.73 **±** 0.18 | Tachi et al. (2015) |
| Chemotherapy rather than HRT, not receiving radiotherapy |  | Public (w) | 46 | 46 | TTO | 0.46 | Milne et al. (2006) |
|  |  | Public (w) | 50 | 46 | EQ-5D UK | 0.48 | Milne et al. (2006) |
|  |  | Public (w) | 47 | 46 | EQ-5D NZ | 0.49 | Milne et al. (2006) |
|  |  | Public (w) | 50 | 46 | VAS | 0.51 | Milne et al. (2006) |

Tachi et al. (2015) - values are not given by type of chemotherapy regimen. 36.4% of patients were administered a regimen of epirubicin plus cyclophosphamide, 53% of participants had adjuvant chemotherapy; Mansel et al (2007) - most patients were on Tamoxifen;

**indicates median (interquartile range); ABC, Advanced Breast Cancer; EBC, Early Breast Cancer; HRT, Hormone Replacement Therapy; BC, Breast Cancer; (w), women only; EQ-5D, Euro-QOL 5D; VAS, Visual Analogue Scale; TTO, Time Trade Off; SG, Standard Gamble; QOL-VAS, Quality of Life-Visual Analogue Scale

- 1. **Chemotherapy – drug specified**

| Health state | | Respondents | Sample size | Age in years (mean, mean ± standard deviation is provided when available) | Health utility estimation method | Health Utility value (mean, mean ± standard deviation when available | Study |
| --- | --- | --- | --- | --- | --- | --- | --- |
| 5-Fluorouracil, Doxorubicin, Cyclophosphamide (FAC) | Third cycle | Patients | 48 | 44.7 ± 8.5 | EQ-5D | 0.75 ± 0.18 | Conner-Spady et al. (2005) |
|  | Third cycle | Patients | 48 | 44.7 ± 8.5 | QOL-VAS | 0.77 ± 0.04 | Conner-Spady et al. (2005) |
|  | Last session | Patients | 68 | 49.29 ± 11.59 | 15D | 0.70 | Bastani et al. (2012) |
|  | Last session | Patients | 68 | 49.29 ± 11.59 | EQ-5D | 0.64 | Bastani et al. (2012) |
|  | 4 months post | Patients | 68 | 49.29 ± 11.59 | 15D | 0.75 | Bastani et al. (2012) |
|  | 4 months post | Patients | 68 | 49.29 ± 11.59 | EQ-5D | 0.73 | Bastani et al. (2012) |
| Docetaxel with Doxorubicin and Cyclophosphamide (TAC) | Last session | Patients | 32 | 46.71 ± 8.23 | 15D | 0.66 | Bastani et al. (2012) |
|  | Last session | Patients | 32 | 46.71 ± 8.23 | EQ-5D | 0.55 | Bastani et al. (2012) |
|  | 4 months post | Patients | 32 | 46.71 ± 8.23 | 15D | 0.76 | Bastani et al. (2012) |
|  | 4 months post | Patients | 32 | 46.71 ± 8.23 | EQ-5D | 0.73 | Bastani et al. (2012) |
| Standard dose chemotherapy - Doxorubicin or Epirubicin and Cyclophosphamide followed by classical CMF | During CT | Patients | 149 | 25% < 40 | SHE | 0.60 | Bernhard et al. (2008) |
|  | During CT | Patients | 149 | 25% < 40 | TTO | 0.77 | Bernhard et al. (2008) |
|  | Toxicity from adjuvant treatment | Patients | 27 | 25% < 40 | SHE | 0.51 | Bernhard et al. (2008) |
|  | Toxicity from adjuvant treatment | Patients | 27 | 25% < 40 | TTO | 0.68 | Bernhard et al. (2008) |
|  | No adverse events | Patients | 140 | 25% < 40 | SHE | 0.80 | Bernhard et al. (2008) |
|  | No adverse events | Patients | 140 | 25% < 40 | TTO | 0.92 | Bernhard et al. (2008) |
|  | Relapse | Patients | 51 | 25% < 40 | SHE | 0.55 | Bernhard et al. (2008) |
|  | Relapse | Patients | 51 | 25% < 40 | TTO | 0.72 | Bernhard et al. (2008) |
| Dose-intensive Epirubicin Cyclophosphamide followed by dose intensive Doxorubcin and Cyclophosphamide | During CT | Patients | 135 | 25% < 40 | SHE | 0.57 | Bernhard et al. (2008) |
|  | During CT | Patients | 135 | 25% < 40 | TTO | 0.74 | Bernhard et al. (2008) |
|  | Toxicity from adjuvant treatment | Patients | 69 | 25% < 40 | SHE | 0.55 | Bernhard et al. (2008) |
|  | Toxicity from adjuvant treatment | Patients | 69 | 25% < 40 | TTO | 0.72 | Bernhard et al. (2008) |
|  | No adverse events | Patients | 152 | 25% < 40 | SHE | 0.77 | Bernhard et al. (2008) |
|  | No adverse events | Patients | 152 | 25% < 40 | TTO | 0.90 | Bernhard et al. (2008) |
|  | Relapse | Patients | 34 | 25% < 40 | SHE | 0.64 | Bernhard et al. (2008) |
|  | Relapse | Patients | 34 | 25% < 40 | TTO | 0.80 | Bernhard et al. (2008) |
| FAC followed by MVC HDC with ASCT support | 6 months | Patients | 45 | 44.7 ± 8.5 | EQ-5D | 0.79 ± 0.19 | Conner-Spady et al. (2005) |
|  | 6 months | Patients | 45 | 44.7 ± 8.5 | QOL-VAS | 0.80 ± 0.04 | Conner-Spady et al. (2005) |
|  | 12 months | Patients | 40 | 44.7 ± 8.5 | EQ-5D | 0.84 ± 0.19 | Conner-Spady et al. (2005) |
|  | 12 months | Patients | 40 | 44.7 ± 8.5 | QOL-VAS | 0.83 ± 0.04 | Conner-Spady et al. (2005) |
|  | 24 months | Patients | 37 | 44.7 ± 8.5 | EQ-5D | 0.89 ± 0.13 | Conner-Spady et al. (2005) |
|  | 24 months | Patients | 37 | 44.7 ± 8.5 | QOL-VAS | 0.89 ± 0.03 | Conner-Spady et al. (2005) |
| Paclitaxel | 3 cycles | Patients | 75 | 42.7% - 50- <60 | EQ-5D | 0.78 | Shiroiwa et al. (2011) |
|  | 5 cycles | HCP | 24 | 44.9 | TTO | 0.36** | Dranitsaris et al. (2009) |
|  | 5 cycles | Patients | 28 | 50 | TTO | 0.57** | Dranitsaris et al. (2015) |
|  | Post-treatment | Patients | 83 | 60.5* | EQ-5D | 0.66 ± 0.25 | Fountzilas et al. (2009) |
|  | 6 months | Patients | 72 | 60.5* | EQ-5D | 0.74 ± 0.22 | Fountzilas et al. (2009) |
|  | 1 year (8 cycles) | Patients | 75 | 42.7% - 50- <60 | EQ-5D | 0.80 | Shiroiwa et al. (2011) |
| Anthracycline followed by paclitaxel | 3 cycles | Patients | 74 | 41% - 50- <60 | EQ-5D | 0.80 | Shiroiwa et al. (2011) |
|  | 1 year (8 cycles) | Patients | 74 | 41% - 50- <60 | EQ-5D | 0.85 | Shiroiwa et al. (2011) |
| Docetaxel | 3 cycles | Patients | 75 | 37.3% - 50- <60 | EQ-5D | 0.80 | Shiroiwa et al. (2011) |
|  | 6 cycles | HCP | 24 | 44.9 | TTO | 0.44** | Dranitsaris et al. (2009) |
|  | 6 cycles | Patients | 28 | 50 | TTO | 0.51** | Dranitsaris et al. (2015) |
|  | 1 year (8 cycles) | Patients | 75 | 37.3% - 50- <60 | EQ-5D | 0.79 | Shiroiwa et al. (2011) |
| Anthracycline-containing regimens followed by docetaxel | 3 cycles | Patients | 74 | 38% - 50- <60 | EQ-5D | 0.84 | Shiroiwa et al. (2011) |
|  | 1 year (8 cycles) | Patients | 74 | 38% - 50- <60 | EQ-5D | 0.85 | Shiroiwa et al. (2011) |
| Nab-paclitaxel | 6 cycles | HCP | 24 | 44.9 | TTO | 0.51** | Dranitsaris et al. (2009) |
|  | 6 cycles | Patients | 28 | 50 | TTO | 0.63** | Dranitsaris et al. (2015) |
| Paclitaxel+ carboplatin (PCb) | Post-treatment | Patients | 78 | 60** | EQ-5D | 0.68 ± 0.22 | Fountzilas et al. (2009) |
|  | 6 months | Patients | 74 | 60** | EQ-5D | 0.70 ± 0.27 | Fountzilas et al. (2009) |
| Gemcitabine+ docetaxel (GDoc) | Post-treatment | Patients | 73 | 60** | EQ-5D | 0.65 ± 0.21 | Fountzilas et al. (2009) |
|  | 6 months | Patients | 62 | 60** | EQ-5D | 0.69 ± 0.23 | Fountzilas et al. (2009) |
| Capecitabine | Treatment response | Patients | 54 | 52** | HUI3 | 0.77 | Reed et al. (2009) |
|  | Stable disease | Patients | 175 | 52** | HUI3 | 0.62 | Reed et al. (2009) |
|  | Grade 3/4 toxicity prior to disease progression | Patients | 17 | 51** | EQ-5D | 0.59 | Sherrill et al. (2008) |
|  | Time spent with grade3/4 toxicity | Patients | 157 | 51** | EQ-5D | 0.66 | Sherrill et al. (2008) |
|  | Disease progression | Patients | 102 | 52** (25-79) | HUI3 | 0.68 | Reed et al. (2009) |
|  | Relapse | Patients | 67 | 51** | EQ-5D | 0.44 | Sherrill et al. (2008) |
|  | Unknown response | Patients | 46 | 52** (25-79) | HUI3 | 0.58 | Reed et al. (2009) |
|  |  | Patients | 168 | 51 | EQ-5D | 0.64 ± 0.26 | Zhou et al. (2009) |
| Ixabepilone Plus Capecitabine | Treatment response | Patients | 130 | 53 (25-76)** | HUI3 | 0.67 | Reed et al. (2009) |
|  | Stable disease | Patients | 155 | 53 (25-76)** | HUI3 | 0.65 | Reed et al. (2009) |
|  | Disease progression | Patients | 58 | 53 (25-76)** | HUI3 | 0.61 | Reed et al. (2009) |
|  | Unknown response | Patients | 32 | 53 (25-76)** | HUI3 | 0.28 | Reed et al. (2009) |
| Lapatinib and Capecitanib | Grade 3/4 toxicity prior to disease progression | Patients | 27 | 54 (26-80)** | EQ-5D | 0.60 | Sherrill et al. (2008) |
|  | Time spent with grade 3/4 toxicity | Patients | 168 | 54 (26-80)** | EQ-5D | 0.66 | Sherrill et al. (2008) |
|  | Relapse | Patients | 50 | 54 (26-80)** | EQ-5D | 0.41 | Sherrill et al. (2008) |
|  |  | Patients | 163 | 54 | EQ-5D | 0.66 ± 0.24 | Zhou et al. (2009) |
| Taxane (paclitaxel or docetaxel) | Progression free survival up to 12 months | Patients | 175 | 57.0 (33–75) | EQ-5D | 0.77 | Shirowa et al. (2016) |
| S-1 | Progression free survival up to 12 months | Patients | 208 | 59.0 (29–75) | EQ-5D | 0.81 | Shirowa et al. (2016) |

** median values; HCP, Health Care Professionals; EQ-5D, Euro-QOL 5D; VAS, Visual Analogue Scale; TTO, Time Trade Off; SG, Standard Gamble; QOL-VAS, Quality of Life-Visual Analogue Scale; HUI3, Health Utilities Index Mark 3; SHE, Subjective Health Estimation

- 1. **Endocrine therapy**

| Health state | | Respondents | Sample size | Age in years (mean, mean ± standard deviation is provided when available) | Health utility estimation method | Health Utility value (mean, mean ± standard deviation when available | Study |
| --- | --- | --- | --- | --- | --- | --- | --- |
| On Hormone replacement therapy |  | Public (w) - ABC | 46 | 46 | TTO | 0.54 | Milne et al. (2006) |
|  |  | Public (w) - ABC | 50 | 46 | VAS | 0.60 | Milne et al. (2006) |
|  |  | Public (w) - ABC | 50 | 46 | EQ-5D (UK) | 0.54 | Milne et al. (2006) |
|  |  | Public (w) - ABC | 40 | 46 | EQ-5D(NZ) | 0.65 | Milne et al. (2006) |
|  |  | Public (w) | 156 | 46.2% ≥ 50 | TTO | 0.54 | Schleinitz et al. (2006) |
|  |  | Patients | 330 | 65** | EQ-5D | 0.86 | Yagata et al. (2016) |
|  |  | Public (w) | 131 | 50-79 | VAS | 0.52 ± 0.22 | Bonomi et al. (2008) |
|  | Within ≤ 6m of diagnosis | Patients | 297 | 57 ± 10.7 | EQ-5D | 0.76 | Hall et al. (2015) |
|  | Within <12m of diagnosis | Patients | 297 | 57 ± 10.7 | EQ-5D | 0.80 | Hall et al. (2015) |
|  | Within >15m of diagnosis | Patients | 297 | 57 ± 10.7 | EQ-5D | 0.79 | Hall et al. (2015) |
|  | First year after primary diagnosis | Patients | 17 | 57 | EQ-5D | 0.74 | Lidgren et al. (2007) |
|  |  | Patients | 17 | 57 | TTO | 0.89 | Lidgren et al. (2007) |
|  | At least 1 recurrence within 1 year of primary BC | Patients | 4 | 57 | EQ-5D | 0.82 | Lidgren et al. (2007) |
|  |  | Patients | 4 | 57 | TTO | 0.86 | Lidgren et al. (2007) |
|  | Second and following years after primary BC | Patients | 79 | 57 | EQ-5D | 0.82 | Lidgren et al. (2007) |
|  |  | Patients | 76 | 57 | TTO | 0.93 | Lidgren et al. (2007) |
|  | For distant recurrence | Patients | 16 | 57 | EQ-5D | 0.65 | Lidgren et al. (2007) |
|  |  | Patients | 17 | 57 | TTO | 0.86 | Lidgren et al. (2007) |
|  |  | Patients | 23 | 68 | SG | 0.88 ± 0.11 | Mansel et al. (2007) |
| Goserelin therapy |  | Patients | 152 | 42.6 ± 7.3 | SG | 0.81 ± 0.17 | Cheng et al. (2012) |
| Tamoxifen |  | Public (w) - BRCA+ | 83 | 67.5% - 35-60 | TTO | 0.95 ± 0.14 | Grann et al. (2010) |
|  |  | Public (w) | 60 | 43.1% - 18-34 | TTO | 0.90 ± 0.16 | Grann et al. (2010) |
|  |  | Public (w) | 219 | 43.8% - 65-74 | SG | 0.75 ± 0.31 | Melnikow et al. (2008) |

BC, breast cancer; (w), women only; BRCA, BReast CAncer gene; ABC, advanced breast cancer; EQ-5D, Euro-QOL 5D; VAS, Visual Analogue Scale; TTO, Time Trade Off; SG, Standard Gamble; VR-6D, Veterans RAND-6D

**Table 4. Health utility values for allied health and complementary medicine**

| Health state | | Respondents | Sample size | Age in years (mean, mean ± standard deviation is provided when available) | Health utility estimation method | Health Utility value (mean, mean ± standard deviation when available | Study |
| --- | --- | --- | --- | --- | --- | --- | --- |
| Psychological support - by oncology nurse | Baseline | Patients | 55 | 55 | EQ-5D | 0.60 ± 0.24 | Arving et al. (2014) |
|  | 24m | Patients | 55 | 55 | EQ-5D | 0.86 ± 0.16 | Arving et al. (2014) |
| Psychological support - by psychologists | Baseline | Patients | 57 | 55 | EQ-5D | 0.64 ± 0.23 | Arving et al. (2014) |
|  | 24m | Patients | 57 | 55 | EQ-5D | 0.81 ± 0.23 | Arving et al. (2014) |
| Psychological support - standard care ± referral to psychiatrist/social worker if deemed necessary | Baseline | Patients | 168 | 55 | EQ-5D | 0.56 ± 0.25 | Arving et al. (2014) |
|  | 24m | Patients | 168 | 55 | EQ-5D | 0.76 ± 023 | Arving et al. (2014) |
| Exercise program – face-to-face or phone - 16 sessions by a trained  and qualified exercise physiologist | Before | Patients | 67 | 52 ± 8 | EQ-5D | 0.79 | Gordon et al (2017) |
|  | 6 months | Patients | 67 | 52 ± 8 | EQ-5D | 0.83 | Gordon et al (2017) |
|  | 12 months | Patients | 67 | 52 ± 8 | EQ-5D | 0.86 | Gordon et al (2017) |
| Guided imagery | Before | Patients | 64 | 57 | EQ-5D | 0.88 ± 0.12 | Serra et al. (2012) |
|  | After | Patients | 54 | 57 | EQ-5D | 0.86 ± 0.10 | Serra et al. (2012) |
| Mindfulness based stress reduction | Baseline | Patients | 19 | 37-65 | EQ-5D | 0.74 | Eyles et al. (2015) |
|  | Week 24 | Patients | 19 | 37-65 | EQ-5D | 0.72 | Eyles et al. (2015) |
| Usual care/maintain habitual activity |  | Patients | 208 | 55 ± 10.3 | SHE | 0.73 ± 0.17 | Gordon et al. (2005) |
|  | Baseline | Patients | 78 | 49.4 ± 7.6 | EQ-5D | 0.87 ± 0.13 | May et al. (2017) |
|  | First 18 weeks | Patients | 78 | 49.4 ± 7.6 | EQ-5D | 0.83 ±0.12 | May et al. (2017) |
|  | Last 18 weeks | Patients | 78 | 49.4 ± 7.6 | EQ-5D | 0.80 ± 0.14 | May et al. (2017) |
|  | Before | Patients | 60 | 52 ± 8 | EQ-5D | 0.83 | Gordon et al (2017) |
|  | 6 months | Patients | 60 | 52 ± 8 | EQ-5D | 0.81 | Gordon et al (2017) |
|  | 12 months | Patients | 60 | 52 ± 8 | EQ-5D | 0.85 | Gordon et al (2017) |
| Water exercising - community | Baseline | Patients | 29 | 42 - 82 | EQ-5D | 0.80 (0.73-1.0)** | Enblom et al. (2017) |
| Shoulder mobility, education, tailored exercise program – community |  | Patients | 36 | 59 ± 10.7 | SHE | 0.77 ± 0.19 | Gordon et al. (2005) |
| Shoulder mobility, education, psychosocial issues – breast clinics |  | Patients | 31 | 54 ± 11.3 | SHE | 0.79 ± 0.18 | Gordon et al. (2005) |
| Aerobic and strength program – outpatient clinic | Baseline | Patients | 87 | 50 ± 7.9 | EQ-5D | 0.88 ± 0.13 | May et al. (2017) |
|  | First 18 weeks | Patients | 87 | 50 ± 7.9 | EQ-5D | 0.83 ± 0.14 | May et al. (2017) |
|  | Last 18 weeks | Patients | 87 | 50 ± 7.9 | EQ-5D | 0.82 ± 0.13 | May et al. (2017) |
| Multimedia instructional package and home-based strength, balance, shoulder mobility, and cardiovascular endurance program - home | Baseline | Patients | 45 | 55.9 ± 10.5 | EQ-5D | 0.81 ± 0.14 | Haines et al. (2010) |
|  | 3 months | Patients | 36 | 55.9 ± 10.5 | EQ-5D | 0.78 ± 0.19 | Haines et al. (2010) |
|  | 6 months | Patients | 35 | 55.9 ± 10.5 | EQ-5D | 0.80 ± 0.21 | Haines et al. (2010) |
| Multimedia instructional package and static stretching program - home | Baseline | Patients | 42 | 54.2 ± 11.5 | EQ-5D | 0.85 ± 0.19 | Haines et al. (2010) |
|  | 3 months | Patients | 37 | 54.2 ± 11.5 | EQ-5D | 0.84 ± 0.17 | Haines et al. (2010) |
|  | 6 months | Patients | 34 | 54.2 ± 11.5 | EQ-5D | 0.83 ± 0.18 | Haines et al. (2010) |
| Others* - Not shown in figures | | | | | | | |
| Short-stay admission program | One day before surgery | Patients | 127 | 56.1 ± 10.8 | EQ-5D | 0.84 ± 0.02 | de Kok et al. (2010) |
| Usual length of stay | One day before surgery | Patients | 135 | 55.3 ± 11.6 | EQ-5D | 0.80 ± 0.02 | de Kok et al. (2010) |
| Follow-up after breast cancer treatment completed – usual care |  | Patients | 74 | 57.2 ± 9.8 | EQ-5D | 0.74 ± 0.23 | Kimman et al (2011) |
| Follow-up after breast cancer treatment completed with nurse |  | Patients | 76 | 55.5 ± 9.0 | EQ-5D | 0.73 ± 0.21 | Kimman et al (2011) |
| Follow-up as usual after breast cancer treatment completed and educational group program |  | Patients | 75 | 55.3 ± 11.5 | EQ-5D | 0.80 ± 0.18 | Kimman et al (2011) |
| Follow-up after breast cancer treatment completed with nurse and educational group program |  | Patients | 74 | 55.4 ± 9.2 | EQ-5D | 0.73 ± 0.23 | Kimman et al (2011) |

**median values (interquartile range); EQ-5D, Euro-QOL 5D; SHE, Subjective Health Estimation

**Table 5: Health state utility values by adverse events**

| Type of adverse event | Severity grade++  (when available) | Respondents | Sample size | Age in years (mean, mean ± standard deviation is provided when available) | Health utility estimation method | Health Utility value (mean, mean ± standard deviation when available | Study |
| --- | --- | --- | --- | --- | --- | --- | --- |
| No side effect, no recurrence |  | HCP | 20 | 33.2 ± 6 | VAS | 0.87 | Shih et al. (2012) |
|  |  | HCP | 20 | 33.2 ± 6 | SG | 0.68 | Shih et al. (2012) |
|  |  | Patients | 35 | 50.1 ± 8.2 | VAS | 0.82 ± 0.14 | Tan et al. (2014) |
|  |  | Patients | 33 | 50.1 ± 8.2 | SG | 0.80 ± 0.22 | Tan et al. (2014) |
| Common adverse events – Tamoxifen |  | Patients | 26 | 68 | SG | 0.97 ± 0.04 | Mansel et al. (2007) |
| Common adverse events – Anastrozole |  | Patients | 26 | 68 | SG | 0.96 ± 0.06 | Mansel et al. (2007) |
| Fatigue |  | Public - Sweden | 100 | 48% - 18-29 | TTO | 0.64 ± 0.30 | Frederix et al. (2013) |
|  |  | Public - Netherlands | 100 | 51% - 50-59 | TTO | 0.56 ± 0.27 | Frederix et al. (2013) |
|  | Grade 1-2 | Patients | 69 | 54 ± 10.7 | SG | 0.72 ± 0.21 | Kuchuk et al. (2013) |
|  | Grade 3-4 | Patients | 69 | 54 ± 10.7 | SG | 0.72 ± 0.18 | Kuchuk et al. (2013) |
|  |  | General public | 100 | 40.16 ± 13.59 | SG | 0.60 | Lloyd et al. (2006) |
|  | Grade 0 | Patients | 21 | 59.6 ± 12.2 | EQ-5D | 0.78 ± 0.14 | Tachi et al. (2015) |
|  | Grade 1-3 | Patients | 26 | 59.6 ± 12.2 | EQ-5D | 0.69 ± 0.21 | Tachi et al. (2015) |
| Anorexia |  | Public - Sweden | 100 | 48% - 18-29 | TTO | 0.56 ± 0.30 | Frederix et al. (2013) |
|  |  | Public - Netherlands | 100 | 51% - 50-59 | TTO | 0.66 ± 0.24 | Frederix et al. (2013) |
|  | Grade 0 | Patients | 27 | 59.6 ± 12.2 | EQ-5D | 0.77 ± 0.16 | Tachi et al. (2015) |
|  | Grade 1-3 | Patients | 20 | 59.6 ± 12.2 | EQ-5D | 0.68 ± 0.21 | Tachi et al. (2015) |
| Nausea | Grade 1-2 | Patients | 69 | 54 ± 10.7 | SG | 0.73 ± 13.0 | Kuchuk et al. (2013) |
|  | Grade 3-4 | Patients | 69 | 54 ± 10.7 | SG | 0.62 ± 22.2 | Kuchuk et al. (2013) |
|  | Grade 0 | Patients | 35 | 59.6 ± 12.2 | EQ-5D | 0.72 ± 0.20 | Tachi et al. (2015) |
|  | Grade 1-3 | Patients | 12 | 59.6 ± 12.2 | EQ-5D | 0.74 ± 0.12 | Tachi et al. (2015) |
| Diarrhea |  | Public - Sweden | 100 | 48% - 18-29 | TTO | 0.52 ± 0.31 | Frederix et al. (2013) |
|  |  | Public - Netherlands | 100 | 51% - 50-59 | TTO | 0.50 ± 0.25 | Frederix et al. (2013) |
|  | Grade 1-2 | Patients | 69 | 54 ± 10.7 | SG | 0.76 ± 16.8 | Kuchuk et al. (2013) |
|  | Grade 3-4 | Patients | 69 | 54 ± 10.7 | SG | 0.68 ± 22.1 | Kuchuk et al. (2013) |
| Diarrhea and vomiting |  | Public | 100 | 40.16 ± 13.59 | SG | 0.61 | Lloyd et al. (2006) |
| Constipation | Grade 0 | Patients | 31 | 59.6 ± 12.2 | EQ-5D | 0.76 ± 0.14 | Tachi et al. (2015) |
|  | Grade 1-3 | Patients | 16 | 59.6 ± 12.2 | EQ-5D | 0.67 ± 0.24 | Tachi et al. (2015) |
| Oral pain | Grade 0 | Patients | 42 | 59.6 ± 12.2 | EQ-5D | 0.74 ± 0.19 | Tachi et al. (2015) |
|  | Grade 1-3 | Patients | 5 | 59.6 ± 12.2 | EQ-5D | 0.66 ± 0.15 | Tachi et al. (2015) |
| Altered taste | Grade 0 | Patients | 39 | 59.6 ± 12.2 | EQ-5D | 0.72 ± 0.19 | Tachi et al. (2015) |
|  | Grade 1-3 | Patients | 8 | 59.6 ± 12.2 | EQ-5D | 0.77 ± 0.12 | Tachi et al. (2015) |
| Alopecia |  | Patients | 69 | 54 ± 10.7 | SG | 0.72 ± 22.5 | Kuchuk et al. (2013) |
|  |  | Public | 100 | 40.16 ± 13.59 | SG | 0.60 | Lloyd et al. (2006) |
|  | Grade 0 | Patients | 16 | 59.6 ± 12.2 | EQ-5D | 0.76 ± 0.18 | Tachi et al. (2015) |
|  | Grade 1-3 | Patients | 31 | 59.6 ± 12.2 | EQ-5D | 0.71 ± 0.19 | Tachi et al. (2015) |
| Lymphedema |  | Patients | 128 | 16-89 | EQ-5D | 0.80 ± 0.02 | Cheville (2010) |
| Anemia |  | Public - Sweden | 100 | 48% - 18-29 | TTO | 0.69 ± 0.29 | Frederix et al. (2013) |
|  |  | Public - Netherlands | 100 | 51% - 50-59 | TTO | 0.59 ± 0.26 | Frederix et al. (2013) |
|  | Grade 0 | Patients | 29 | 59.6 ± 12.2 | EQ-5D | 0.74 ± 0.16 | Tachi et al. (2015) |
|  | Grade 1-3 | Patients | 18 | 59.6 ± 12.2 | EQ-5D | 0.70 ± 0.22 | Tachi et al. (2015) |
| Febrile neutropenia |  | Public | 100 | 40.16 ± 13.59 | SG | 0.57 | Lloyd et al. (2006) |
|  | Grade 0 | Patients | 40 | 59.6 ± 12.2 | EQ-5D | 0.74 ± 0.19 | Tachi et al. (2015) |
|  | Grade 1-3 | Patients | 7 | 59.6 ± 12.2 | EQ-5D | 0.69 ± 0.21 | Tachi et al. (2015) |
| Reduced WBC count | Grade 0 | Patients | 37 | 59.6 ± 12.2 | EQ-5D | 0.71 ± 0.19 | Tachi et al. (2015) |
|  | Grade 1-3 | Patients | 10 | 59.6 ± 12.2 | EQ-5D | 0.81 ± 0.14 | Tachi et al. (2015) |
|  |  | Public - Sweden | 100 | 48% - 18-29 | TTO | 0.58 ± 0.31 | Frederix et al. (2013) |
|  |  | Public - Netherlands | 100 | 51% - 50-59 | TTO | 0.60 ± 0.26 | Frederix et al. (2013) |
| Skin rash |  | Public - Sweden | 100 | 48% - 18-29 | TTO | 0.58 ± 0.31 | Frederix et al. (2013) |
|  |  | Public - Netherlands | 100 | 51% - 50-59 | TTO | 0.54 ± 0.27 | Frederix et al. (2013) |
| Nail ridging | Grade 0 | Patients | 42 | 59.6 ± 12.2 | EQ-5D | 0.73 ± 0.19 | Tachi et al. (2015) |
|  | Grade 1-3 | Patients | 5 | 59.6 ± 12.2 | EQ-5D | 0.68 ± 0.15 | Tachi et al. (2015) |
| Dry skin | Grade 0 | Patients | 40 | 59.6 ± 12.2 | EQ-5D | 0.74 ± 0.18 | Tachi et al. (2015) |
|  | Grade 1-3 | Patients | 7 | 59.6 ± 12.2 | EQ-5D | 0.64 ± 0.16 | Tachi et al. (2015) |
| Decrease in left ventricular ejection fraction |  | Public - Sweden | 100 | 48% - 18-29 | TTO | 0.54 ± 0.29 | Frederix et al. (2013) |
|  |  | Public - Netherlands | 100 | 51% - 50-59 | TTO | 0.95 ± 0.25 | Frederix et al. (2013) |
| Hand-foot syndrome | Grade 1-2 | Patients | 69 | 54 ± 10.7 | SG | 0.75 ± 16.7 | Kuchuk et al. (2013) |
|  | Grade 3-4 | Patients | 69 | 54 ± 10.7 | SG | 0.70 ± 18.9 | Kuchuk et al. (2013) |
|  |  | Public | 100 | 40.16 ± 13.59 | SG | 0.60 | Lloyd et al. (2006) |
| Mucositis | Grade 0 | Patients | 35 | 59.6 ± 12.2 | EQ-5D | 0.74 ± 0.19 | Tachi et al. (2015) |
|  | Grade 1-3 | Patients | 12 | 59.6 ± 12.2 | EQ-5D | 0.68 ± 0.17 | Tachi et al. (2015) |
| Stomatitis |  | Public (w) | 100 | 40.16 ± 13.59 | SG | 0.56 | Lloyd et al. (2006) |
| Mucositis/stomatitis | Grade 1-2 | Patients | 69 | 54 ± 10.7 | SG | 0.75 ± 17.9 | Kuchuk et al. (2013) |
|  | Grade 3-4 | Patients | 69 | 54 ± 10.7 | SG | 0.74 ± 17.9 | Kuchuk et al. (2013) |
| Sensory neuropathy | Grade 1-2 | Patients | 69 | 54 ± 10.7 | SG | 0.73 ± 18.9 | Kuchuk et al. (2013) |
|  | Grade 3-4 | Patients | 69 | 54 ± 10.7 | SG | 0.69 ± 19.1 | Kuchuk et al. (2013) |
|  | Grade 0 | Patients | 38 | 59.6 ± 12.2 | EQ-5D | 0.74 ± 0.18 | Tachi et al. (2015) |
|  | Grade 1-3 | Patients | 9 | 59.6 ± 12.2 | EQ-5D | 0.67 ± 0.18 | Tachi et al. (2015) |
| Motor neuropathy | Grade 1-2 | Patients | 69 | 54 ± 10.7 | SG | 0.72 ± 14.5 | Kuchuk et al. (2013) |
|  | Grade 3-4 | Patients | 69 | 54 ± 10.7 | SG | 0.73 ± 15.1 | Kuchuk et al. (2013) |
| Deep vein thromboembolism |  | Patients - Tamoxifen | 26 | 68 | SG | 0.92 ± 0.11 | Mansel et al. (2007) |
|  |  | Public (w) - no history of BC | 32 | 43.8% - 65-74 | SG | 0.66 ± 0.42 | Melnikow et al. (2008) |
|  |  | HCP | 20 | 33.2 ± 6 | VAS | 0.58 | Shih et al. (2012) |
|  |  | HCP | 20 | 33.2 ± 6 | SG | 0.52 | Shih et al. (2012) |
|  |  | Patients | 31 | 50.1 ± 8.2 | VAS | 0.45 ± 0.20 | Tan et al. (2014) |
|  |  | Patients | 29 | 50.1 ± 8.2 | SG | 0.56 ± 0.25 | Tan et al. (2014) |
| Pulmonary embolism |  | Patients - Tamoxifen | 26 | 68 | SG | 0.89 ± 0.17 | Mansel et al. (2007) |
|  |  | Public (w) - no history of BC | 30 | 43.8% - 65-74 | SG | 0.50 ± 0.39 | Melnikow et al. (2008) |
|  |  | HCP | 20 | 33.2 ± 6 | VAS | 0.37 | Shih et al. (2012) |
|  |  | HCP | 20 | 33.2 ± 6 | SG | 0.46 | Shih et al. (2012) |
|  |  | Patients | 31 | 50.1 ± 8.2 | VAS | 0.26 ± 0.27 | Tan et al. (2014) |
|  |  | Patients | 29 | 50.1 ± 8.2 | SG | 0.40 ± 0.40 | Tan et al. (2014) |
| Myalgia | Grade 1-2 | Patients | 69 | 54 ± 10.7 | SG | 0.72 ± 14.5 | Kuchuk et al. (2013) |
|  | Grade 3-4 | Patients | 69 | 54 ± 10.7 | SG | 0.70 ± 13.8 | Kuchuk et al. (2013) |
| Musculoskeletal disorder |  | HCP | 20 | 33.2 ± 6 | VAS | 0.65 | Shih et al. (2012) |
|  |  | HCP | 20 | 33.2 ± 6 | SG | 0.51 | Shih et al. (2012) |
|  |  | Patients | 35 | 50.1 ± 8.2 | VAS | 0.41 ± 0.34 | Tan et al. (2014) |
|  |  | Patients | 34 | 50.1 ± 8.2 | SG | 0.45 ± 0.38 | Tan et al. (2014) |
| Osteoporotic fracture |  | Public (w) - no history of BC | 33 | 43.8% - 65-74 | SG | 0.78 ± 0.28 | Melnikow et al. (2008) |
| Wrist fracture |  | Patients - Tamoxifen | 26 | 68 | SG | 0.92 ± 0.10 | Mansel et al. (2007) |
|  |  | HCP | 20 | 33.2 ± 6 | VAS | 0.51 | Shih et al. (2012) |
|  |  | HCP | 20 | 33.2 ± 6 | SG | 0.53 | Shih et al. (2012) |
|  |  | Patients | 36 | 50.1 ± 8.2 | VAS | 0.44 ± 0.23 | Tan et al. (2014) |
|  |  | Patients | 33 | 50.1 ± 8.2 | SG | 0.53 ± 0.18 | Tan et al. (2014) |
| Spinal fracture |  | Patients - Tamoxifen | 26 | 68 | SG | 0.89 ± 0.19 | Mansel et al. (2007) |
|  |  | HCP | 20 | 33.2 ± 6 | VAS | 0.41 | Shih et al. (2012) |
|  |  | HCP | 20 | 33.2 ± 6 | SG | 0.46 | Shih et al. (2012) |
|  |  | Patients | 40 | 50.1 ± 8.2 | VAS | 0.26 ± 0.51 | Tan et al. (2014) |
|  |  | Patients | 37 | 50.1 ± 8.2 | SG | 0.32 ± 0.29 | Tan et al. (2014) |
| Hip fracture |  | Patients - Tamoxifen | 26 | 68 | SG | 0.86 ± 0.20 | Mansel et al. (2007) |
|  |  | HCP | 20 | 33.2 ± 6 | VAS | 0.51 | Shih et al. (2012) |
|  |  | HCP | 20 | 33.2 ± 6 | SG | 0.50 | Shih et al. (2012) |
|  |  | Patients | 35 | 50.1 ± 8.2 | VAS | 0.33 ± 0.23 | Tan et al. (2014) |
|  |  | Patients | 34 | 50.1 ± 8.2 | SG | 0.43 ± 0.36 | Tan et al. (2014) |
| Cataracts |  | Public (w) - no history of BC | 31 | 43.8% - 65-75 | SG | 0.77 ± 0.31 | Melnikow et al. (2008) |
|  |  | HCP | 20 | 33.2 ± 6 | VAS | 0.55 | Shih et al. (2012) |
|  |  | HCP | 20 | 33.2 ± 6 | SG | 0.52 | Shih et al. (2012) |
|  |  | Patients | 31 | 50.1 ± 8.2 | VAS | 0.37 ± 0.39 | Tan et al. (2014) |
|  |  | Patients | 28 | 50.1 ± 8.2 | SG | 0.43 ± 0.32 | Tan et al. (2014) |
| Hot flushes |  | HCP | 20 | 33.2 ± 6 | VAS | 0.75 | Shih et al. (2012) |
|  |  | HCP | 20 | 33.2 ± 6 | SG | 0.59 | Shih et al. (2012) |
|  |  | Patients | 36 | 50.1 ± 8.2 | VAS | 0.60 ± 0.19 | Tan et al. (2014) |
|  |  | Patients | 33 | 50.1 ± 8.2 | SG | 0.63 ± 0.21 | Tan et al. (2014) |
| Vaginal bleeding |  | Patients - Tamoxifen | 26 | 68 | SG | 0.93 ± 0.10 | Mansel et al. (2007) |
|  |  | HCP | 20 | 33.2 ± 6 | VAS | 0.73 | Shih et al. (2012) |
|  |  | HCP | 20 | 33.2 ± 6 | SG | 0.55 | Shih et al. (2012) |
|  |  | Patients | 35 | 50.1 ± 8.2 | VAS | 0.58 ± 0.23 | Tan et al. (2014) |
|  |  | Patients | 33 | 50.1 ± 8.2 | SG | 0.65 ± 0.25 | Tan et al. (2014) |
| Hysterectomy |  | Patients - Tamoxifen | 26 | 68 | SG | 0.90 ± 0.10 | Mansel et al. (2007) |
| Severe bone pain requiring radiotherapy |  | Public (w) - no history of BC | 50 | 46 | VAS | 0.25 | Milne et al. (2006) |
|  |  | Public (w) - no history of BC | 50 | 46 | EQ-5D (UK) | 0.31 | Milne et al. (2006) |
|  |  | Public (w) - no history of BC | 45 | 46 | EQ-5D (NZ) | 0.45 | Milne et al. (2006) |
|  |  | Public (w) - no history of BC | 46 | 46 | TTO | 0.35 | Milne et al. (2006) |
| Moderate to severe hypercalcaemia |  | Public (w) - no history of BC | 50 | 46 | VAS | -0.52 | Milne et al. (2006) |
|  |  | Public (w) - no history of BC | 50 | 46 | EQ-5D (UK) | -0.05 | Milne et al. (2006) |
|  |  | Public (w) - no history of BC | 50 | 46 | EQ-5D (NZ) | -0.17 | Milne et al. (2006) |
|  |  | Public (w) - no history of BC | 46 | 46 | TTO | 0.13 | Milne et al. (2006) |
| Ischemic cerebrovascular events |  | HCP | 20 | 33.2 ± 6 | VAS | 0.30 | Shih et al. (2012) |
|  |  | HCP | 20 | 33.2 ± 6 | SG | 0.41 | Shih et al. (2012) |
|  |  | Patients | 35 | 50.1 ± 8.2 | VAS | 0.25 ± 0.30 | Tan et al. (2014) |
|  |  | Patients | 34 | 50.1 ± 8.2 | SG | 0.35 ± 0.26 | Tan et al. (2014) |
| Endometrial cancer |  | Patients - Tamoxifen | 26 | 68 | SG | 0.91 ± 0.10 | Mansel et al. (2007) |
|  |  | Public(w) - no history of BC | 20 | 43.8% - 65-74 | SG | 0.59 ± 0.39 | Melnikow et al. (2008) |
|  |  | HCP | 20 | 33.2 ± 6 | VAS | 0.51 | Shih et al. (2012) |
|  |  | HCP | 20 | 33.2 ± 6 | SG | 0.50 | Shih et al. (2012) |
|  |  | Patients | 35 | 50.1 ± 8.2 | VAS | 0.40 ± 0.22 | Tan et al. (2014) |
|  |  | Patients | 34 | 50.1 ± 8.2 | SG | 0.43 ± 0.40 | Tan et al. (2014) |
| New contralateral breast cancer |  | Patients - Tamoxifen | 26 | 68 | SG | 0.91 ± 0.10 | Mansel et al. (2007) |
|  |  | HCP | 20 | 33.2 ± 6 | VAS | 0.50 | Shih et al. (2012) |
|  |  | HCP | 20 | 33.2 ± 6 | SG | 0.44 | Shih et al. (2012) |
|  |  | Patients | 35 | 50.1 ± 8.2 | VAS | 0.46 ± 0.25 | Tan et al. (2014) |
|  |  | Patients | 34 | 50.1 ± 8.2 | SG | 0.45 ± 0.32 | Tan et al. (2014) |
| Local/regional recurrence with no adverse events |  | Patients - Tamoxifen | 26 | 68 | SG | 0.91 ± 0.10 | Mansel et al. (2007) |
|  |  | HCP | 20 | 33.2 ± 6 | VAS | 0.56 | Shih et al. (2012) |
|  |  | HCP | 20 | 33.2 ± 6 | SG | 0.47 | Shih et al. (2012) |
|  |  | Patients | 37 | 50.1 ± 8.2 | VAS | 0.34 ± 0.21 | Tan et al. (2014) |
|  |  | Patients | 35 | 50.1 ± 8.2 | SG | 0.46 ± 0.30 | Tan et al. (2014) |
| Local/regional recurrence with adverse events |  | HCP | 20 | 33.2 ± 6 | VAS | 0.51 | Shih et al. (2012) |
|  |  | HCP | 20 | 33.2 ± 6 | SG | 0.44 | Shih et al. (2012) |
| Distant recurrence with no adverse effects |  | HCP | 20 | 33.2 ± 6 | VAS | 0.44 | Shih et al. (2012) |
|  |  | HCP | 20 | 33.2 ± 6 | SG | 0.47 | Shih et al. (2012) |
|  |  | Patients | 32 | 50.1 ± 8.2 | VAS | 0.34 ± 0.19 | Tan et al. (2014) |
|  |  | Patients | 31 | 50.1 ± 8.2 | SG | 0.42 ± 0.28 | Tan et al. (2014) |
| Distant recurrence with adverse effects ± chemotherapy |  | Patients | 38 | 50.1 ± 8.2 | VAS | 0.30 ± 0.30 | Tan et al. (2014) |
|  |  | Patients | 34 | 50.1 ± 8.2 | SG | 0.31 ± 0.40 | Tan et al. (2014) |
|  |  | HCP | 20 | 33.2 ± 6 | VAS | 0.40 | Shih et al. (2012) |
|  |  | HCP | 20 | 33.2 ± 6 | SG | 0.46 | Shih et al. (2012) |
| Distant recurrence with adverse effects ± hormonal therapy |  | Patients | 31 | 50.1 ± 8.2 | VAS | 0.30 ± 0.24 | Tan et al. (2014) |
|  |  | Patients | 29 | 50.1 ± 8.2 | SG | 0.33 ± 0.28 | Tan et al. (2014) |
|  |  | HCP | 20 | 33.2 ± 6 | VAS | 0.41 | Shih et al. (2012) |
|  |  | HCP | 20 | 33.2 ± 6 | SG | 0.45 | Shih et al. (2012) |

++For definition of the severity grade, please refer to the published article; BC, Breast Cancer; HCP, Health Care Provider; VAS, Visual Analogue Scale; SG, Standard Gamble; TTO, Time Trade Off

**Table 5: Health utility values by the stage of breast cancer**

1. **Early breast cancer**

| Health state | | Respondents | Sample size | Age in years (mean, mean ± standard deviation is provided when available) | Health utility estimation method | Health Utility value (mean, mean ± standard deviation when available | Study |
| --- | --- | --- | --- | --- | --- | --- | --- |
| Stage 1 |  | Public (w) | 156 | 46.2% ≥ 50 | TTO | 0.68 | Schleinitz et al. (2006) |
| Stage 2 |  | Public (w) | 156 | 46.2% ≥ 50 | TTO | 0.61 | Schleinitz et al. (2006) |
| Diagnosis ≤ 6m |  | Patients | 297 | 57 ± 10.7 | EQ-5D | 0.76 | Hall et al. (2015) |
| Diagnosis <12m |  | Patients | 297 | 57 ± 10.7 | EQ-5D | 0.78 | Hall et al. (2015) |
| Diagnosis >15m |  | Patients | 297 | 57 ± 10.7 | EQ-5D | 0.79 | Hall et al. (2015) |
| Disease free | no adverse events | Patients | 26 | 68 | SG | 0.99 ± 0.01 | Mansel et al. (2007) |
|  | first year | Patients | 2684 | - | EQ-5D | 0.73 (SE = 0.02) | Seferina et al. (2017) |
|  | > first year | Patients | 2684 | - | EQ-5D | 0.81 (SE = 0.02) | Seferina et al. (2017) |
| Remission/Pre-relapse |  | Patients | 929 | 49** | EQ-5D | 0.79 ± 0.02 | Wolowacz et al. (2008) |
| Local recurrence | first year | Patients | 2684 | - | EQ-5D | 0.73 (SE = 0.02) | Seferina et al. (2017) |
|  | > first year | Patients | 2684 | - | EQ-5D | 0.71 (SE = 0.09) | Seferina et al. (2017) |
| Distant metastasis | first year | Patients | 2684 | - | EQ-5D | 0.58 (SE = 0.06) | Seferina et al. (2017) |
|  | > first year | Patients | 2684 | - | EQ-5D | 0.60 (SE = 0.05) | Seferina et al. (2017) |

**median values; SG, Standard Gamble

1. **Advanced/Metastatic breast cancer**

| Health state | | Respondents | Sample size | Age in years (mean, mean ± standard deviation is provided when available) | Health utility estimation method | Health Utility value (mean, mean ± standard deviation when available | Study |
| --- | --- | --- | --- | --- | --- | --- | --- |
| Advanced/Metastatic breast cancer |  | Patients | 27 | 66 ± 10.4 | 15D | 0.72 ± 0.14 | Farkkila et al. (2014) |
|  |  | Patients | 27 | 66 ± 10.4 | EQ-5D | 0.45 ± 0.37 | Farkkila et al. (2014) |
|  | Sweden | Public | 100 | 18-29 - 48% | TTO | 0.81 ± 0.23 | Frederix et al. (2013) |
|  | The Netherlands | Public | 100 | 50-59 - 51% | TTO | 0.69 ± 0.25 | Frederix et al. (2013) |
|  | Stage 4 | Public | 509 | 45.7 ± 14.10 | VAS | 0.17 ± 0.22 | Kim et al. (2017) |
|  | Stage 4 | Public | 509 | 45.7 ± 14.10 | SG | 0.35 ± 0.28 | Kim et al. (2017) |
|  | Stage 3C | Public | 509 | 45.7 ± 14.10 | VAS | 0.42 ± 0.17 | Kim et al. (2017) |
|  | Stage 3C | Public | 509 | 45.7 ± 14.10 | SG | 0.59 ± 0.26 | Kim et al. (2017) |
|  |  | Public | 100 | 40.16 ± 13.59 | SG | 0.72 | Lloyd et al. (2006) |
|  |  | Patients | 61 | 57 | TTO | 0.82 | Lidgren et al. (2007) |
|  |  | Patients | 65 | 57 | EQ-5D | 0.69 | Lidgren et al. (2007) |
|  |  | Patients | 188 | 49.8 ± 9.89 | EQ-5D | 0.54 - 0.62 | Oh et al. (2012) |
|  |  | Patients | 58 | 62 (36-85)** | EQ-5D | 0.66 (SE=0.05) | van Kampen (2017) |
|  |  | Patients | 24 | 46.7± 9.97 | SF-6D | 0.59 ± 0.13 | Yousefi et al. (2016) |
|  |  | Patients | 24 | 46.7 ± 9.97 | EQ-5D | 0.55 ± 0.23 | Yousefi et al. (2016) |
|  | Stage 3 | Public (w) | 156 | 46.2% ≥ 50 | TTO | 0.56 | Schleinitz et al. (2006) |
|  | Stage 4- | Public (w) | 156 | 46.2% ≥ 50 | TTO | 0.42 | Schleinitz et al. (2006) |
|  | Stage 4+ | Public (w) | 156 | 46.2% ≥ 50 | TTO | 0.41 | Schleinitz et al. (2006) |
| On treatment |  | Patients | 52 | 54 ± 11.20 | EQ-5D | 0.75 ± 0.17 | Pickard et al. (2016) |
|  |  | Patients | 15 | 56.9 ± 14 | EQ-5D | 0.69 ± 0.26 | Wallwiener et al. (2017) |
| Responding to treatment |  | Public | 100 | 40.16 ± 13.59 | SG | 0.08 | Lloyd et al. (2006) |
| Progressive disease |  | Public - Sweden | 100 | 18-29 - 48% | TTO | 0.61 ± 0.34 | Frederix et al. (2013) |
|  |  | Public - Netherlands | 100 | 50-59 - 51% | TTO | 0.49 ± 0.31 | Frederix et al. (2013) |
|  |  | Public | 100 | - 1. ± 13.59 | SG | 0.44 | Lloyd et al. (2006) |
|  | Local recurrence | Public (w) | 110 | 26 – 60 | SG | 0.61 (SE=0.03) | Songtish et al.(2014) |
|  | Local recurrence with lymphedema | Public (w) | 110 | 26 – 60 | SG | 0.39 (SE=0.03) | Songtish et al.(2014) |
|  | Regional recurrence | Public (w) | 110 | 26 – 60 | SG | 0.60 (SE=0.04) | Songtish et al.(2014) |
|  | Regional recurrence with lymphedema | Public (w) | 110 | 26 – 60 | SG | 0.45 (SE=0.03) | Songtish et al.(2014) |
|  |  | Patients | 38 | 62 (36-85)** | EQ-5D | 0.55 (SE = 0.08) | van Kampen et al. (2017) |
| Last year of life |  | Patients | 150 | 65+ - 49.2% | HALex | 0.64 | Yabroff et al. (2007) |

**median values; SG, Standard Gamble; TTO, Time Trade Off; VAS, Visual Analogue Scale; HALex, Health Activity Limitations Index

1. **Non-specific breast cancer**

| Health state | Respondents | Sample size | Age in years (mean, mean ± standard deviation is provided when available) | Health utility estimation method | Health Utility value (mean, mean ± standard deviation when available | Study |
| --- | --- | --- | --- | --- | --- | --- |
| Breast cancer | Public (w) | 259 | 18-44 | EQ-5D (US) | 0.89 | Brown et al (2016) |
|  | Public (w) | 7174 | ≥ 45 | EQ-5D (US) | 0.82 | Brown et al (2016) |
|  | Patients | 41 | 51.6 | AQOL-4D | 0.59 | Garvey et al. (2016) |
|  | Public (w) - BRCA+ | 83 | 67.5% between 35-60 | TTO | 0.87 ± 0.20 | Grann et al. (2010) |
|  | Public (w) | 160 | 43.1% 18-34 | TTO | 0.84 ± 0.18 | Grann et al. (2010) |
|  | Patients | 2445 | 51.8 | EQ-5D | 0.68 ± 0.18 | Kimman et al. (2015) |
|  | Patients | 608 | 48 ± 9.6 | EQ-5D | 0.84 ± 0.15 | Liu et al. (2017) |
|  | Patients | 608 | 48 ± 9.6 | EQ-5D (China) | 0.83 ± 0.18 | Liu et al. (2017) |
|  | Patients | 608 | 48 ± 9.6 | EQ-5D (Korea) | 0.83 ± 0.14 | Liu et al. (2017) |
|  | Patients | 608 | 48 ± 9.6 | EQ-5D (Japan) | 0.80 ± 0.16 | Liu et al. (2017) |
|  | Public (w) | 45 | 43.1% - 18-34 | SG | 0.57 ± 0.38 | Melnikow et al. (2008) |
|  | Patients | 287 | 59 | EQ-5D | 0.76 ± 0.24 | Naik et al. (2017) |
|  | Patients | 287 | 59 | EQ-5D (CAN) | 0.80 ± 0.17 | Naik et al. (2017) |
|  | Patients | 287 | 59 | EQ-5D (US) | 0.82 ± 0.17 | Naik et al. (2017) |
|  | Patients | 59 | 51 | SF-6D | 0.81 ± 0.12 | Shih et al. (2006) |
|  | Patients | 201 | 64 | EQ-5D | 0.80 | Sullivan et al. (2006) |
|  | Patients | 385 | 64.2 | EQ-5D | 0.75 | Sullivan et al. (2011) |
|  | Patients | 7426 | 85% ≥45 | EQ-5D | 0.79 ± 0.18 | Trogdon et al. (2016) |
| Initial diagnosis | Patients | 14 |  | VAS | 0.56** | Lux et al. (2010) |
|  | Patients | 389 | 49.2% ≥ 65 | HALex | 0.78 | Yabroff et al. (2007) |
| Disease free at 1 year/Unremarkable in follow-up | Public | 131 | 65% < 64 | VAS | 0.77 ± 0.13 | Bonomi et al. (2008) |
|  | Patients | 42 |  | VAS | 0.72** | Lux et al. (2010) |
| First year after primary breast cancer | Patients | 1654 | 52 | EQ-5D | 0.76 ± 0.21 | The ACTION Study Group (2017) |
|  | Patients | 69 | 57 | TTO | 0.90 | Lidgren et al. (2007) |
|  | Patients | 72 | 57 | EQ-5D | 0.70 | Lidgren et al. (2007) |
|  | Patients | 104 | 59 | EQ-5D | 0.80 ± 0.02 | Naik et al. (2017) |
|  | Patients | 48 | 46.7 ± 9.97 | EQ-5D | 0.67 ± 0.20 | Yousefi et al. (2016) |
|  | Patients | 48 | 46.7 ± 9.97 | SF-6D | 0.64 ± 0.13 | Yousefi et al. (2016) |
| First year after recurrence | Patients | 21 | 57 | EQ-5D | 0.78 | Lidgren et al. (2007) |
|  | Patients | 18 | 57 | TTO | 0.84 | Lidgren et al. (2007) |
|  | Patients | 15 | 46.7 ± 9.97 | EQ-5D | 0.72 ± 0.14 | Yousefi et al. (2016) |
|  | Patients | 15 | 46.7 ± 9.97 | SF-6D | 0.68 ± 0.06 | Yousefi et al. (2016) |
| Second and following years post primary breast cancer or recurrence | Public | 131 | 50-79 | VAS | 0.33 ± 0.19 | Bonomi et al. (2008) |
|  | Patients | 178 | 57 | TTO | 0.89 | Lidgren et al. (2007) |
|  | Patients | 177 | 57 | EQ-5D | 0.78 | Lidgren et al. (2007) |
|  | Patients | 179 | 59 | EQ-5D | 0.81 ± 0.01 | Naik et al. (2017) |
|  | Patients | 71 | 46.7 ± 9.97 | EQ-5D | 0.73 ± 0.22 | Yousefi et al. (2016) |
|  | Patients | 71 | 46.7 ± 9.97 | SF-6D | 0.68 ± 0.13 | Yousefi et al. (2016) |
| Recurrence | Patients | 17 |  | VAS | 0.54** | Lux et al. (2010) |
| Survivors | Patients | 150 | 52.8 ± 11.1 | EQ-5D | 0.71 ± 0.25 | Matalqah et al. (2011) |
| In remission, <2 years | Patients | 66 | 14.1% ≥ 80 | SF-6D | 0.60 | Wang et al. (2016) |
| In remission, 2-4 years | Patients | 129 | 14.1% ≥ 80 | SF-6D | 0.62 | Wang et al. (2016) |
| In remission, 5-9 years | Patients | 190 | 14.1% ≥ 80 | SF-6D | 0.64 | Wang et al. (2016) |
| In remission, ≥10 years | Patients | 315 | 14.1% ≥ 80 | SF-6D | 0.63 | Wang et al. (2016) |
| Not in remission | Patients | 48 | 14.1% ≥ 80 | SF-6D | 0.60 | Wang et al. (2016) |
| Continuing care | Patients | 381 | 49.2% ≥ 65 | HALex | 0.81 | Yabroff et al. (2007) |
| Loco-regional recurrent | Public | 509 | 45.7 ± 14.1 | SG | 0.50 ± 0.26 | Kim et al. (2017) |
|  | Public | 509 | 45.7 ± 14.1 | VAS | 0.33 ± 0.18 | Kim et al. (2017) |
| Contralateral breast cancer | Patients | 4 |  | VAS | 0.55** | Lux et al. (2010) |
| Palliation/end of life | Public | 131 | 50-79 | VAS | 0.36 ± 0.27 | Bonomi et al. (2008) |

**median values; SG, Standard Gamble; TTO, Time Trade Off; VAS, Visual Analogue Scale; HALex, Health Activity Limitations Index; SF-6D, Short-form-6D; AQOL-4D, Australian Quality of Life-4D; CAN, Canada; US, United States

**SUPPLEMENTARY MATERIALS REFERENCES (in alphabetical order)**

1. Ali AA, Xiao H, Tawk R, Campbell E, Semykina A, Montero AJ, et al. Comparison of health utility weights among elderly patients receiving breast-conserving surgery plus hormonal therapy with or without radiotherapy. Curr Med Res Opin. 2017;33(2):391-400.
2. Arving C, Brandberg Y, Feldman I, Johansson B, Glimelius B. Cost–utility analysis of individual psychosocial support interventions for breast cancer patients in a randomized controlled study. Psychooncology. 2014;23(3):251-8.
3. Bastani P, Kiadaliri AA. Cost-utility analysis of adjuvant therapies for breast cancer in Iran. Int J Technol Assess Health Care. 2012;28(2):110-4.
4. Bernhard J, Zahrieh D, Zhang J, Martinelli G, Basser R, Hürny C, et al. Quality of life and quality-adjusted survival (Q-TWiST) in patients receiving dose-intensive or standard dose chemotherapy for high-risk primary breast cancer. Br J Cancer. 2008;98(1):25.
5. Bonomi AE, Boudreau DM, Fishman PA, Ludman E, Mohelnitzky A, Cannon EA, et al. Quality of life valuations of mammography screening. Qual Life Res. 2008;17(5):801-14.
6. Brown DS, Trogdon JG, Ekwueme DU, Chamiec-Case L, Guy Jr GP, Tangka FK, et al. Health state utility impact of breast cancer in US women aged 18–44 years. Am J Prev Med. 2016;50(2):255-61
7. Cheng TF, Der Wang J, Uen WC. Cost-utility analysis of adjuvant goserelin (Zoladex) and adjuvant chemotherapy in premenopausal women with breast cancer. BMC Cancer. 2012;12(1):33.
8. Cheville AL, Almoza M, Courmier JN, Basford JR. A prospective cohort study defining utilities using time trade‐offs and the Euroqol‐5D to assess the impact of cancer‐related lymphedema. Cancer. 2010;116(15):3722-31.
9. Conner-Spady B, Cumming C, Nabholtz J, Jacobs P, Stewart D. A longitudinal prospective study of health-related quality of life in breast cancer patients following high-dose chemotherapy with autologous blood stem cell transplantation. Bone Marrow Transplant. 2005;36(3):251.
10. Diane Serra R, MSL A, Parris CR, Elise Carper R, Fleishman SB, Harrison LB, et al. Outcomes of guided imagery in patients receiving radiation therapy for breast cancer. Clin J Oncol Nurs. 2012;16(6):617.
11. de Kok M, Dirksen CD, Kessels AG, van der Weijden T, van de Velde CJ, Roukema JA, et al. Cost-effectiveness of a short stay admission programme for breast cancer surgery. Acta Oncol. 2010;49(3):338-46.
12. Domeyer PJ, Sergentanis TN, Zagouri F, Zografos GC. Health-related quality of life in vacuum-assisted breast biopsy: short-term effects, long-term effects and predictors. Health Qual Life Outcomes. 2010;8(1):11.
13. Dranitsaris G, Cottrell W, Spirovski B, Hopkins S. Economic analysis of albumin-bound paclitaxel for the treatment of metastatic breast cancer. J Oncol Pharm Pract. 2009;15(2):67-78.
14. Dranitsaris G, Yu B, King J, Kaura S, Zhang A. Nab-paclitaxel, docetaxel, or solvent-based paclitaxel in metastatic breast cancer: a cost-utility analysis from a Chinese health care perspective. Clinicoecon Outcomes Res. 2015;7:249.
15. Enblom A, Lindquist H, Bergmark K. Participation in water‐exercising long‐term after breast cancer surgery: Experiences of significant factors for continuing exercising as a part of cancer rehabilitation. Eur J Cancer care. 2017.
16. Eyles C, Leydon GM, Hoffman CJ, Copson ER, Prescott P, Chorozoglou M, et al. Mindfulness for the self-management of fatigue, anxiety, and depression in women with metastatic breast cancer: a mixed methods feasibility study. Integr Cancer Ther. 2015;14(1):42-56.
17. Farkkila N, Torvinen S, Roine RP, Sintonen H, Hänninen J, Taari K, et al. Health-related quality of life among breast, prostate, and colorectal cancer patients with end-stage disease. Qual Life Res. 2014;23(4):1387-94.
18. Frederix GW, Quadri N, Hövels AM, van de Wetering FT, Tamminga H, Schellens JH, et al. Utility and work productivity data for economic evaluation of breast cancer therapies in the Netherlands and Sweden. Clin Ther. 2013;35(4):e1-e7.
19. Freedman GM, Li T, Anderson PR, Nicolaou N, Konski A. Health states of women after conservative surgery and radiation for breast cancer. Breast Cancer Res Treat. 2010;121(2):519-26.
20. Fountzilas G, Dafni U, Dimopoulos M, Koutras A, Skarlos D, Papakostas P, et al. A randomized phase III study comparing three anthracycline-free taxane-based regimens, as first line chemotherapy, in metastatic breast cancer. Breast Cancer Res Treat. 2009;115(1):87.
21. Garvey G, Cunningham J, He VY, Janda M, Baade P, Sabesan S, et al. Health-related quality of life among Indigenous Australians diagnosed with cancer. Qual Life Res. 2016;25(8):1999-2008.
22. Gordon LG, Scuffham P, Battistutta D, Graves N, Tweeddale M, Newman B. A cost-effectiveness analysis of two rehabilitation support services for women with breast cancer. Breast Cancer Res Treat. 2005;94(2):123-33.
23. Gordon LG, DiSipio T, Battistutta D, Yates P, Bashford J, Pyke C, et al. Cost‐effectiveness of a pragmatic exercise intervention for women with breast cancer: results from a randomized controlled trial. Psychooncology. 2017;26(5):649-55.
24. Grann VR, Patel P, Bharthuar A, Jacobson JS, Warner E, Anderson K, et al. Breast cancer-related preferences among women with and without BRCA mutations. Breast Cancer Res Treat. 2010;119(1):177.
25. Group Action Study. Health-related quality of life and psychological distress among cancer survivors in Southeast Asia: results from a longitudinal study in eight low-and middle-income countries. BMC Med. 2017;15(1):10.
26. Haines TP, Sinnamon P, Wetzig NG, Lehman M, Walpole E, Pratt T, et al. Multimodal exercise improves quality of life of women being treated for breast cancer, but at what cost? Randomized trial with economic evaluation. Breast Cancer Res Treat. 2010;124(1):163-75.
27. Hall P, Hamilton P, Hulme C, Meads D, Jones H, Newsham A, et al. Costs of cancer care for use in economic evaluation: a UK analysis of patient-level routine health system data. Br J Cancer. 2015;112(5):948.
28. Hayman JA, Kabeto MU, Schipper MJ, Bennett JE, Vicini FA, Pierce LJ. Assessing the benefit of radiation therapy after breast-conserving surgery for ductal carcinoma-in-situ. J Clin Oncol. 2005;23(22):5171-7.
29. Humphrey KL, Lee JM, Donelan K, Kong CY, Williams O, Itauma O, et al. Percutaneous breast biopsy: effect on short-term quality of life. Radiol. 2014;270(2):362-8.
30. Kim S-H, Jo M-W, Ock M, Lee H-J, Lee J-W. estimation of health state utilities in breast cancer. Patient Prefer Adher. 2017;11:531.
31. Kimman M, Dirksen C, Voogd A, Falger P, Gijsen BC, Thuring M, et al. Economic evaluation of four follow-up strategies after curative treatment for breast cancer: results of an RCT. Eur J Cancer. 2011;47(8):1175-85.
32. Kimman M, Jan S, Monaghan H, Woodward M. The relationship between economic characteristics and health-related quality of life in newly diagnosed cancer patients in Southeast Asia: results from an observational study. Qual Life Res. 2015;24(4):937-49.
33. Knuttel FM, van den Bosch MA, Young-Afat DA, Emaus MJ, van den Bongard DH, Witkamp AJ, et al. Patient Preferences for Minimally Invasive and Open Locoregional Treatment for Early-Stage Breast Cancer. Value Health. 2017;20(3):474-80.
34. Kuchuk I, Bouganim N, Beusterien K, Grinspan J, Vandermeer L, Gertler S, et al. Preference weights for chemotherapy side effects from the perspective of women with breast cancer. Breast Cancer Res Treat. 2013;142(1):101-7.
35. Lidgren M, Wilking N, Jönsson B, Rehnberg C. Health related quality of life in different states of breast cancer. Qual Life Res. 2007;16(6):1073-81.
36. Liu L, Li S, Wang M, Chen G. Comparison of eQ-5D-5l health state utilities using four country-specific tariffs on a breast cancer patient sample in mainland china. Patient Prefer Adher. 2017;11:1049.
37. Lloyd A, Nafees B, Narewska J, Dewilde S, Watkins J. Health state utilities for metastatic breast cancer. Br J Cancer. 2006;95(6):683.
38. Lux MP, Reichelt C, Wallwiener D, Kreienberg R, Jonat W, Gnant M, et al. Results of the Zometa® cost-utility model for the german healthcare system based on the results of the ABCSG-12 study. Oncol Res Treat. 2010;33(7):360-8.
39. Mansel R, Locker G, Fallowfield L, Benedict A, Jones D. Cost-effectiveness analysis of anastrozole vs tamoxifen in adjuvant therapy for early stage breast cancer in the United Kingdom: the 5-year completed treatment analysis of the ATAC (‘Arimidex’, Tamoxifen alone or in combination) trial. Br J Cancer. 2007;97(2):152.
40. Matalqah LM, Radaideh KM, Yusoff ZM, Awaisu A. Health-related quality of life using EQ-5D among breast cancer survivors in comparison with age-matched peers from the general population in the state of Penang, Malaysia. J Public Health. 2011;19(5):475.
41. May AM, Bosch MJ, Velthuis MJ, Van Der Wall E, Bisschop CNS, Los M, et al. Cost-effectiveness analysis of an 18-week exercise programme for patients with breast and colon cancer undergoing adjuvant chemotherapy: the randomised PACT study. BMJ Open. 2017;7(3):e012187.
42. Melnikow J, Birch S, Slee C, McCarthy TJ, Helms LJ, Kuppermann M. Tamoxifen for breast cancer risk reduction: impact of alternative approaches to quality-of-life adjustment on cost-effectiveness analysis. Med Care. 2008;46(9):946-53.
43. Milne RJ, Heaton-Brown KH, Hansen P, Thomas D, Harvey V, Cubitt A. Quality-of-life valuations of advanced breast cancer by New Zealand women. Pharmacoeconomics. 2006;24(3):281-92.
44. Min YH, Lee JW, Shin Y-W, Jo M-W, Sohn G, Lee J-H, et al. Daily collection of self-reporting sleep disturbance data via a smartphone app in breast cancer patients receiving chemotherapy: a feasibility study. J Med Internet Res. 2014;16(5).
45. Moro-Valdezate D, Peiró S, Buch-Villa E, Caballero-Gárate A, Morales-Monsalve MD, Martínez-Agulló Á, et al. Evolution of health-related quality of life in breast cancer patients during the first year of follow-up. J Breast Cancer. 2013;16(1):104-11.
46. Moro-Valdezate D, Buch-Villa E, Peiró S, Morales-Monsalve MD, Caballero-Gárate A, Martínez-Agulló Á, et al. Factors associated with health-related quality of life in a cohort of Spanish breast cancer patients. Breast cancer. 2014;21(4):442-52.
47. Naik H, Howell D, Su S, Qiu X, Brown MC, Vennettilli A, et al. EQ-5D Health Utility Scores: Data from a Comprehensive Canadian Cancer Centre. Patient. 2017;10(1):105-15.
48. Oh DY, Crawford B, KIM SB, CHUNG HC, McDonald J, Lee SY, et al. Evaluation of the willingness‐to‐pay for cancer treatment in Korean metastatic breast cancer patients: A multicenter, cross‐sectional study. Asia‐Pacific J Clin Oncol. 2012;8(3):282-91.
49. Pickard AS, Jiang R, Lin H-W, Rosenbloom S, Cella D. Using patient-reported outcomes to compare relative burden of cancer: EQ-5D and functional assessment of cancer therapy-general in eleven types of cancer. Clin Ther. 2016;38(4):769-77.
50. Postma EL, Koffijberg H, Verkooijen H, Witkamp A, van den Bosch M, van Hillegersberg R. Cost-effectiveness of radioguided occult lesion localization (ROLL) versus wire-guided localization (WGL) in breast conserving surgery for nonpalpable breast cancer: results from a randomized controlled multicenter trial. Ann Surg Oncol. 2013;20(7):2219-26.
51. Prescott R, Kunkler I, Williams L, King C, Jack W, van der Pol M, et al. A randomised controlled trial of postoperative radiotherapy following breast-conserving surgery in a minimum-risk older population. The PRIME trial. Health Technol Assess. 2007;11(31):1-170.
52. Reed SD, Li Y, Anstrom KJ, Schulman KA. Cost effectiveness of ixabepilone plus capecitabine for metastatic breast cancer progressing after anthracycline and taxane treatment. J Clin Oncol. 2009;27(13):2185-91.
53. Robertson S, Wengström Y, Eriksen C, Sandelin K. Breast surgeons performing immediate breast reconstruction with implants–assessment of resource-use and patient-reported outcome measures. Breast. 2012;21(4):590-6.
54. Schleinitz MD, DePalo D, Blume J, Stein M. Can differences in breast cancer utilities explain disparities in breast cancer care? J Gen Intern Med. 2006;21(12):1253-60.
55. Seferina SC, Ramaekers BL, Maaike de Boer M, van den Berkmortel F, van Kampen RJ, van de Wouw AJ, et al. Cost and cost-effectiveness of adjuvant trastuzumab in the real world setting: A study of the Southeast Netherlands Breast Cancer Consortium. Oncotarget. 2017;8(45):79223.
56. Sherrill B, Amonkar M, Stein S, Walker M, Geyer C, Cameron D. Q-TWiST analysis of lapatinib combined with capecitabine for the treatment of metastatic breast cancer. Br J Cancer. 2008;99(5):711.
57. Shih Y-CT, Wang XS, Cantor SB, Cleeland CS. The association between symptom burdens and utility in Chinese cancer patients. Qual Life Res. 2006;15(8):1427-38.
58. Shih V, Chan A, Xie F, Ko Y. Health state utility assessment for breast cancer. Value Health Reg Issues. 2012;1(1):93-7.
59. Shiroiwa T, Fukuda T, Shimozuma K, Kuranami M, Suemasu K, Ohashi Y, et al. Comparison of EQ-5D scores among anthracycline-containing regimens followed by taxane and taxane-only regimens for node-positive breast cancer patients after surgery: the N-SAS BC 02 trial. Value Health. 2011;14(5):746-51.
60. Shiroiwa T, Fukuda T, Shimozuma K, Mouri M, Hagiwara Y, Doihara H, et al. Long-term health status as measured by EQ-5D among patients with metastatic breast cancer: comparison of first-line oral S-1 and taxane therapies in the randomized phase III SELECT BC trial. Qual Life Res. 2017;26(2):445-53.
61. Sinno H, Izadpanah A, Thibaudeau S, Christodoulou G, Lin SJ, Dionisopoulos T. An objective assessment of the perceived quality of life of living with bilateral mastectomy defect. Breast. 2013;22(2):168-72.
62. Sinno H, Izadpanah A, Vorstenbosch J, Dionisopoulos T, Ibrahim AM, Tobias AM, et al. Living with a unilateral mastectomy defect: a utility assessment and outcomes study. J Reconstr Microsurg. 2014;30(05):313-8.
63. Songtish D, Praditsitthikorn N, Teerawattananon Y. A Cost-Utility Analysis Comparing Standard Axillary Lymph Node Dissection with Sentinel Lymph Node Biopsy in Patients with Early Stage Breast Cancer in Thailand. Value Health Reg Issues. 2014;3:59-66.
64. Sullivan PW, Ghushchyan V. Preference-based EQ-5D index scores for chronic conditions in the United States. Med Decis Making. 2006;26(4):410-20.
65. Sullivan PW, Slejko JF, Sculpher MJ, Ghushchyan V. Catalogue of EQ-5D scores for the United Kingdom. Med Decis Making. 2011;31(6):800-4.
66. Swan JS, Kong CY, Hur C, Halpern EF, Itauma O, Williams O, et al. Comparing morbidities of testing with a new index: screening colonoscopy versus core-needle breast biopsy. J Am Coll Radiol. 2015;12(3):295-301.
67. Tachi T, Teramachi H, Tanaka K, Asano S, Osawa T, Kawashima A, et al. The impact of outpatient chemotherapy-related adverse events on the quality of life of breast cancer patients. PloS One. 2015;10(4):e0124169.
68. Tan X-Y, Aung M-M, Ngai M-I, Xie F, Ko Y. Assessment of Preference for Hormonal Treatment–Related Health States among Patients with Breast Cancer. Value Health Reg Issues. 2014;3:27-32.
69. Timmers JM, Damen JA, Pijnappel RM, Verbeek AL, den Heeten GJ, Adang EM, et al. Cost-effectiveness of non-invasive assessment in the Dutch breast cancer screening program versus usual care: A randomized controlled trial. Can J Public Health. 2014;105(5):e342e7.
70. Tosteson AN, Fryback DG, Hammond CS, Hanna LG, Grove MR, Brown M, et al. Consequences of false-positive screening mammograms. JAMA Intern Med. 2014;174(6):954-61.
71. Trogdon JG, Ekwueme DU, Chamiec-Case L, Guy GP. Breast Cancer in Young Women. Am J Prev Med. 2016;50(2):262-9.
72. van Kampen R, Ramaekers B, Lobbezoo D, de Boer M, Dercksen M, van den Berkmortel F, et al. Real-world and trial-based cost-effectiveness analysis of bevacizumab in HER2-negative metastatic breast cancer patients: a study of the Southeast Netherlands Breast Cancer Consortium. Eur J Cancer. 2017;79:238-46.
73. Wallwiener M, Heindl F, Brucker SY, Taran F-A, Hartkopf A, Overkamp F, et al. Implementation and feasibility of electronic Patient-Reported Outcome (ePRO) data entry in the PRAEGNANT real-time advanced and metastatic breast cancer registry. Geburtshilfe Frauenheilkd. 2017;77(08):870-8.
74. Wang S-Y, Hsu SH, Gross CP, Sanft T, Davidoff AJ, Ma X, et al. Association between time since cancer diagnosis and health-related quality of life: a population-level analysis. Value Health. 2016;19(5):631-8.
75. Wolowacz SE, Cameron DA, Tate HC, Bagust A. Docetaxel in combination with doxorubicin and cyclophosphamide as adjuvant treatment for early node-positive breast cancer: a cost-effectiveness and cost-utility analysis. J Clin Oncol. 2008;26(6):925-33.
76. Yabroff KR, McNeel TS, Waldron WR, Davis WW, Brown ML, Clauser S, et al. Health limitations and quality of life associated with cancer and other chronic diseases by phase of care. Med Care. 2007:629-37.
77. Yagata H, Ohtsu H, Komoike Y, Saji S, Takei H, Nakamura T, et al. Joint symptoms and health-related quality of life in postmenopausal women with breast cancer who completed 5 years of anastrozole. Support Care Cancer. 2016;24(2):683-9.
78. Yousefi M, Najafi S, Ghaffari S, Mahboub-Ahari A, Ghaderi H. Comparison of SF-6D and EQ-5D Scores in Patients With Breast Cancer. Iran Red Crescent Med J. 2016;18(5).
79. Zhou X, Cella D, Cameron D, Amonkar MM, Segreti A, Stein S, et al. Lapatinib plus capecitabine versus capecitabine alone for HER2+ (ErbB2+) metastatic breast cancer: quality-of-life assessment. Breast Cancer Res Treat. 2009;117(3):577-89.
